# Genomic profiling of *Elizabethkingia anophelis* clinical isolates from a Shanghai hospital: phylogenetic divergence coexists with heterogeneous antibiotic resistance and virulence determinants

**DOI:** 10.3389/fmicb.2025.1751256

**Published:** 2026-01-16

**Authors:** Jiasheng Xiong, Tiantian Han, Jingjing Hu, Yitian Wu, Xiaoyan Huang, Dianyu Yang, Weiwei Hou, Yan Lin

**Affiliations:** 1 Department of Hospital Infection Control, Tongji Hospital, School of Medicine, Tongji University, Shanghai, China; 2 Shanghai Municipal Center for Disease Control and Prevention, Shanghai, China; 3 Department of Epidemiology, School of Public Health, Fudan University, Shanghai, China; 4 Shanghai Institute of Infectious Disease and Biosecurity, Fudan University, Shanghai, China; 5 Innostellar Biotherapeutics Co. Ltd, Shanghai, China; 6 Department of Laboratory Medicine, Tongji Hospital, School of Medicine, Tongji University, Shanghai, China

**Keywords:** antimicrobial resistance, *Elizabethkingia anophelis*, emerging pathogen, phylogenetic analysis, virulence determinants, whole-genome sequencing

## Abstract

**Introduction:**

*Elizabethkingia anophelis* (*E. anophelis*) has emerged as a multidrug-resistant pathogen with limited therapeutic options. This study aimed to characterize antimicrobial resistance mechanisms and virulence determinants in six clinical isolates from Shanghai, China, to inform evidence-based treatment strategies.

**Methods:**

Six strains were isolated from hospitalized patients (five community-acquired, one healthcare-associated) between September–November 2023. Antimicrobial susceptibility testing followed CLSI M100 guidelines. Whole-genome sequencing employed hybrid Illumina/PacBio approaches. Phylogenetic relationships were determined through 16S rRNA gene sequence analysis using the Neighbor-Joining method. Antimicrobial resistance genes and virulence factors were annotated using the Comprehensive Antibiotic Resistance Database (CARD) and Virulence Factor Database (VFDB), with relative gene abundance quantified via a TPM-like (transcripts per million-like) method.

**Results:**

All isolates exhibited resistance to *β*-lactams, fluoroquinolones, carbapenems, and aminoglycosides, but retained minocycline susceptibility (MIC ≤1 μg/mL). Phylogenetic analysis revealed two distinct clusters: Cluster I (EA1, EA3, EA6) aligning with East/Southeast Asian isolates, and Cluster II (EA2, EA4, EA5) showing diverse geographic affinities. Five core resistance mechanisms were identified: antibiotic efflux, antibiotic target alteration, antibiotic inactivation, antibiotic target replacement, and reduced permeability to antibiotics. Virulence determinants included bacterial movement, exotoxin production, biofilm formation, immune regulation, and effector delivery systems. Strain EA5 exhibited unique signatures, including absence of *cesH*, unique *sigE* expression, elevated *AAC(6′)-Iad/aadS* and reduced *qacL/OmpA*.

**Conclusion:**

This study reveals phylogenetically divergent *E. anophelis* lineages in Shanghai with extensive multidrug resistance but preserved minocycline susceptibility. Findings support minocycline-based therapy, enhanced diagnostics, and regional surveillance networks for strain monitoring.

## Graphical abstract

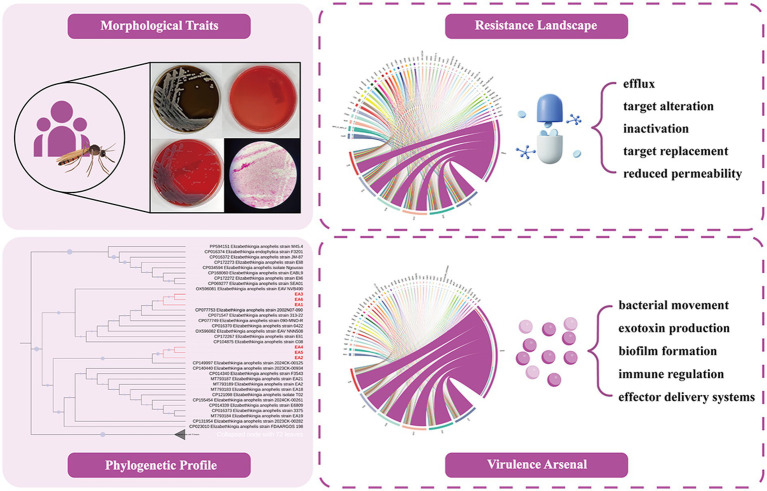

## 1 Introduction

The antimicrobial resistance (AMR) crisis has emerged as one of the most formidable global public health challenges of the 21st century. According to a recent forecast from GBD 2021 Antimicrobial Resistance Collaborators, AMR is projected to directly cause 1.91 million (1.56–2.26 million) deaths and contribute to 8.22 million deaths (6.85–9.65 million) indirectly by 2050 (GBD 2021 Antimicrobial Resistance Collaborators, 2024). The World Health Organization has issued grave warnings that the proliferation of multidrug-resistant pathogens threatens to reverse decades of medical progress, potentially returning clinical practice to a “pre-antibiotic era” (WHO, 2024). Concurrently, advances in molecular diagnostics—particularly whole-genome sequencing and high-throughput identification systems—have revolutionized pathogen identification, revealing a previously underappreciated diversity of opportunistic and emerging bacterial pathogens that possess inherent or acquired resistance mechanisms that substantially complicate clinical management (Li et al., 2021; Nafea et al., 2023).

*Elizabethkingia anophelis* exemplifies this paradigm of emerging AMR pathogens initially overlooked by conventional diagnostics. First described by Kämpfer et al. from laboratory-reared *Anopheles gambiae* mosquitoes (Ifakara strain maintained in Stockholm, Sweden) (Lindh et al., 2008; Kämpfer et al., 2011; Kukutla et al., 2014), this species has rapidly evolved into a clinically significant pathogen with transcontinental spread. Genomic surveillance in China have confirmed its stealthy infiltration, with recent studies documenting multidrug-resistant *E. anophelis* outbreaks in regions like Taizhou City – a sentinel warning of underrecognized transmission networks (Cai et al., 2025). This organism demonstrates extremely ecological adaptability (Wang et al., 2020), capable of both nosocomial transmission via contaminated medical devices or vertical transmission, and community-acquired infections manifesting as bacteremia, meningitis and pneumonia (Lau et al., 2015; Lau et al., 2016). Critically, conventional microbial identification systems frequently misclassify *E. anophelis* as its phenotypically similar relative *E. meningoseptica*, leading to delayed recognition, inappropriate empirical therapies and potentially adverse clinical outcomes (Han et al., 2017).

This study investigates six *E. anophelis* infection cases at Tongji Hospital of Tongji University (Shanghai, China), employing a multi-approaches that combines phenotypic and molecular characterization: (1) standardized isolation and culture protocols to obtain purified strains; (2) broth microdilution susceptibility testing according to CLSI M100 guidelines (34th edition) to evaluate responses to 13 clinically relevant antimicrobial agents; and (3) whole-genome sequencing to elucidate phylogenetic relationships with closely related strains and comparatively inter-strain heterogeneity in resistome and virulence factor repertoires. While recent genomic studies have confirmed intrinsic antimicrobial resistance patterns in *E. anophelis*—including universal minocycline susceptibility (Wu et al., 2024; Wu et al., 2025)—most investigations focused on nosocomial outbreaks or single-center case series. The present study provided genomic characterization of predominantly community-acquired *E. anophelis* infections (5/6 cases) in Shanghai, China, and employed quantitative gene abundance profiling to reveal genomic heterogeneity underlying phenotypically uniform antimicrobial resistance. Our findings underscore the urgent need for enhanced molecular surveillance, targeted antimicrobial stewardship, molecular surveillance of emerging lineages, and tailored therapies to address the evolving resistance and virulence of *E. anophelis*.

## 2 Materials and methods

### 2.1 Study population and clinical data collection

Six patients hospitalized between September and November 2023 with confirmed *E. anophelis* infections were enrolled. Clinical samples, including blood and other secretions, were collected from each patient for bacterial isolation and culture.

Clinical and demographic data were retrieved exclusively from the hospital’s electronic medical records system, including age, sex, underlying comorbidities, primary diagnoses, antibiotic treatment regimens, and clinical outcomes. All specimens were processed according to standard operating procedures to ensure data accuracy and experimental reproducibility.

### 2.2 Pathogen identification and antimicrobial susceptibility testing

Suspected bacterial isolates from clinical samples were cultured on blood agar, chocolate agar, and MacConkey agar plates under aerobic conditions at 35 °C for 24–48 h. After incubation, morphologically typical colonies were selected for Gram staining and microscopic examination. Species identification was confirmed using MALDI-TOF MS (VITEK MS system) with 99.9% confidence scores.

Antimicrobial susceptibility testing was performed by the Broth Microdilution Method to determine minimum inhibitory concentrations (MICs) for *E. anophelis*, in accordance with the Clinical and Laboratory Standards Institute (CLSI) M100 document (34th edition) and non-*Enterobacterales* breakpoints. Plates were incubated aerobically at 35 °C for 16–20 h. *Escherichia coli* ATCC 25922 and *Pseudomonas aeruginosa* ATCC 27853 were included as quality control strains.

The following 13 antimicrobial agents were tested: ciprofloxacin, levofloxacin, piperacillin/tazobactam, ticarcillin/clavulanate, cefoperazone/sulbactam, ceftazidime, cefepime, imipenem, meropenem, amikacin, tobramycin, minocycline, and trimethoprim/sulfamethoxazole.

### 2.3 Sequencing and phylogenetic analysis

Whole-genome sequencing employed combined draft (Illumina PE150) and complete (hybrid Illumina + PacBio) genome approaches. For draft genomes, Illumina PE150 paired-end sequencing with ~400 bp insert size generated ≥100 × coverage per sample. For complete genomes, PacBio long-read sequencing with 8-10 kb fragments provided ≥100 × coverage per sample. Raw reads were quality-filtered using fastp v0.20.0. Draft genomes were assembled using SOAPdenovo v2.04 with GapCloser v1.12 for gap filling, while complete genomes utilized Unicycler for hybrid assembly with Pilon polishing. Gene prediction was performed using Prodigal (chromosomal) and GeneMarkS (plasmid), with tRNA and rRNA identification using tRNAscan-SE v2.0 and Barrnap.

For phylogenetic analysis, 16S rRNA gene sequences were extracted from the six *E. anophelis* genomes. These sequences from the clinical isolates (EA1-EA6) have been deposited in the NCBI GenBank database under accession numbers PV715948–PV715953, respectively. The extracted sequences were used as queries for BLASTn searches against the NCBI GenBank database. For each isolate, the top 50 most similar reference strains and 5 phylogenetically diverse strains were selected based on 16S rRNA sequence similarity. The 16S rRNA sequences from all retrieved reference strains were combined with those of the six study isolates, deduplicated, and aligned using MAFFT v7.490. Phylogenetic trees were constructed using the Neighbor-Joining method in MEGA7.0.26 software.

### 2.4 Analysis of resistance and virulence genes

Antimicrobial resistance genes and virulence factors were identified by the Comprehensive Antibiotic Resistance Database (CARD) and Virulence Factor Database (VFDB), respectively. To enable quantitative comparison of gene relative abundance across isolates, quality-filtered reads were mapped to predicted genes using Bowtie2 v2.4.5. Gene abundance was normalized using a TPM-like (transcripts per million-like) method adapted from metagenomic analysis (Ayala-Muñoz et al., 2020). This normalization adjusts for both gene length and sequencing depth, enabling direct comparison of relative gene abundance between isolates. To identify genes with altered abundance, we calculated the coefficient of variation (CV) for antimicrobial resistance genes and virulence factors separately across the six isolates, yielding CV = 5.4 and 5.7%, respectively. Using the higher CV as a conservative estimate, genes with TPM values ≥ 1.20-fold or ≤ 0.83-fold (1/1.20) relative to the median TPM were classified as having elevated or reduced relative abundance, respectively.

## 3 Results

### 3.1 Clinical management and outcomes

This study enrolled six patients with *E. anophelis* infections admitted between September and November 2023, including one healthcare-associated infection (EA4, diagnosed ≥48 h post-admission per Chinese national criteria) and five community-acquired infections (EA1, EA2, EA3, EA5, EA6, presenting at or before admission). The cohort included four males and two females, with ages ranging from 54 to 90 years (median: 68 years). All patients had ≥2 underlying comorbidities, including predominantly hepatobiliary diseases (4/6), acute infectious complications (4/6), and malignancies (2/6). Specimens were primarily isolated from blood (*n* = 2), bile (*n* = 2), both (*n* = 1), and multiple (*n* = 1). Various comorbidities significantly limited antimicrobial therapeutic options (detailed clinical data see Table 1).

**Table 1:** Clinical data of the six cases of *E. anophelis* infection.

ID	Age	Sex	Specimen	Comorbidities/diagnoses	Antibiotic regimen	Clinical outcome
EA1	54	Male	Bile	Acute suppurative cholecystitis; Gallstones with cholecystitis; Biliary stasis	Cefmetazole sodium (2 g IV Q12H); Cefoperazone-sulbactam (3 g IV Q8H); Ornidazole (1 g IV BID); Doxycycline (100 mg PO Q12H)	Improved
EA2	90	Male	Blood	Severe community-acquired pneumonia; Coronary atherosclerosis; Hypertension grade 3	Meropenem (1 g IV Q8H); Levofloxacin (0.5 g IV QD); Cefoperazone-sulbactam (3 g IV Q8H); Colistin (4 M IU IV Q12H); Vancomycin (0.5 g via tube Q6H)	Unresolved
EA3	65	Male	Blood, Bile	Pancreatic tumor; Hypertension grade 1	Ceftazidime (2 g IV BID); Ornidazole (1 g → 0.5 g IV BID); Cefoperazone-sulbactam (3 g IV Q8H)	Improved
EA4	70	Female	Blood	Multiple fractures; Acute renal failure; Pulmonary infection; Severe anemia	Meropenem (0.5 g IV Q8H); Caspofungin (50 mg IV QD); Colistin (4 M IU IV Q12H); Cefotaxime (1 g IV QD)	Improved
EA5	70	Female	Bile	Obstructive jaundice; Pancreatic malignancy	Cefotaxime (2 g IV BID); Cefmetazole (2 g IV BID; 2 g intraoperative)	Improved
EA6	66	Male	Bile, Pus, Blood, Ascites	Acute biliary pancreatitis; Gallstones with acute cholecystitis; Hypertension grade 2	Imipenem-cilastatin (0.5 g IV Q8H); Cefepime (2 g IV BID); Cefoperazone-sulbactam (3 g IV BID); Metronidazole (500 mg IV BID)	Death

Based on microbiological culture results within 14 days of *E. anophelis* isolation, three cases (EA1, EA3, EA6) were monomicrobial infections, with *E. anophelis* as the sole pathogen isolated from sterile sites (bile, blood, or ascitic fluid). The other three cases (EA2, EA4, EA5) were polymicrobial, where *E. anophelis* was concurrently or sequentially isolated with other pathogens. Notably, in these polymicrobial infections, *E. anophelis* was consistently recovered from sterile compartments (blood or bile), whereas other multidrug-resistant organisms were mainly detected in respiratory or urinary specimens.

Among the four patients with favorable outcomes: EA1 (54-year-old male): *E. anophelis* was isolated from bile. Infection resolved after treatment with doxycycline combined with multiple antibiotics. EA3 (65-year-old male) and EA5 (70-year-old female): Both had pancreatic malignancies with *E. anophelis* confirmed by both blood and bile culture (EA3), and bile (EA5). Clinical improvement was achieved through biliary drainage combined with antibiotics. EA4 (70-year-old female): Hospitalized for multiple fractures, acute renal failure, and pulmonary infection, this healthcare-associated catheter-related infection caused by *E. anophelis* resolved following meropenem and colistin combination therapy.

Two cases exhibited refractory clinical courses: EA2 (90-year-old male): Admitted with sepsis and multi-organ dysfunction (severe pneumonia, coronary heart disease NYHA class IV, lacunar infarction with severe malnutrition). *E. anophelis* was isolated from blood. Despite combined antibiotic regimens, persistent bacteremia led to transfer for palliative long-term care. EA6 (66-year-old male): Hospitalized for acute biliary pancreatitis, *E. anophelis* was detected in multiple clinical specimens. Antimicrobial therapy failed, leading in death due to multiple organ failure.

### 3.2 Pathogen isolation, culture, and antimicrobial susceptibility testing

**Figure 1:**
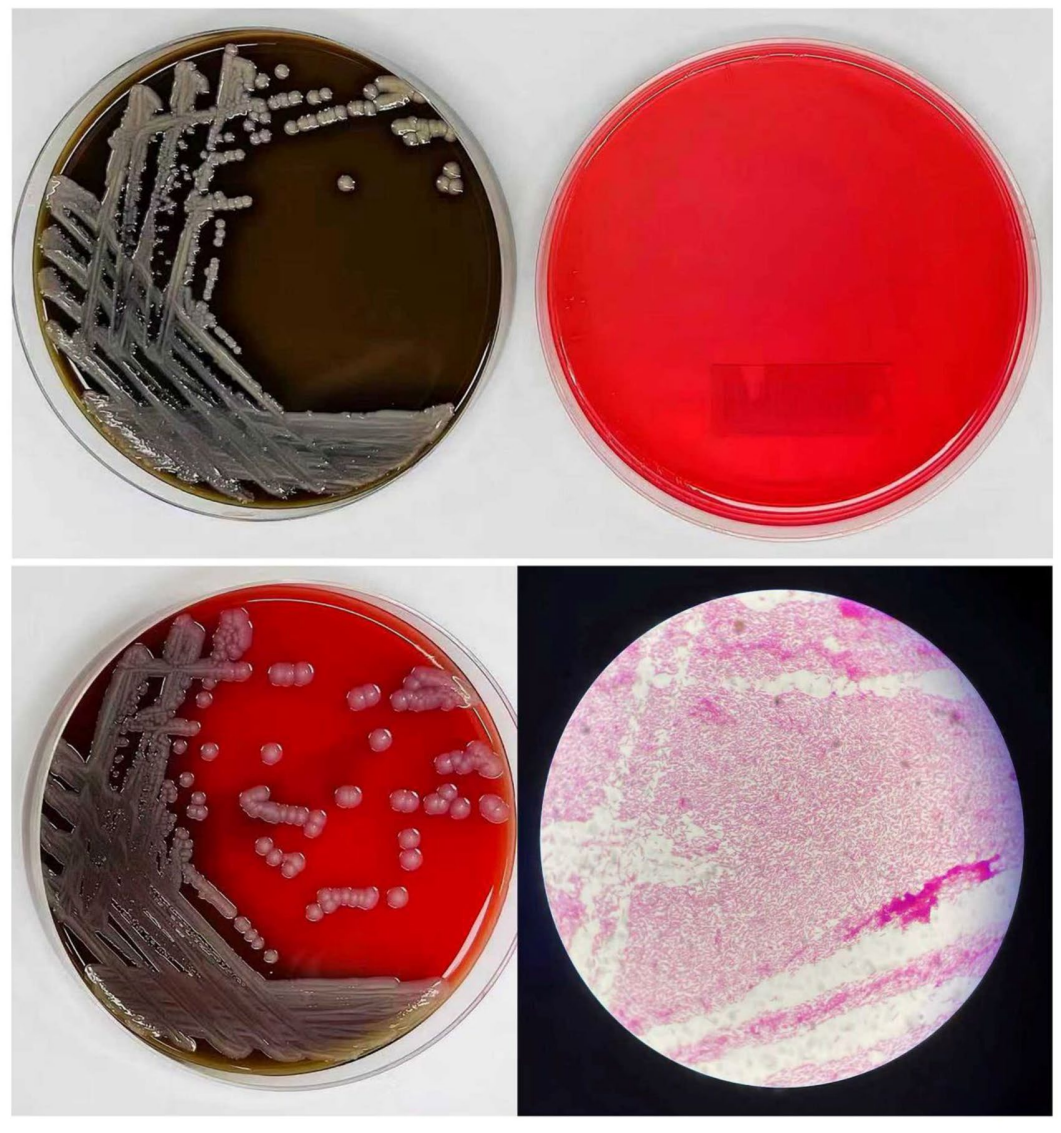
Morphological characteristics of *E. anophelis* (Growth on chocolate agar, MacConkey agar, blood agar, and Gram-stained microscopic image). Representative *E. anophelis* colonies cultured at 35 °C for 48 h under aerobic conditions on different culture media and microscopic examination. Upper left: Chocolate agar showing translucent, circular, gray-white colonies with glossy surface (1–2 mm in diameter). Upper right: MacConkey agar showing no growth, consistent with the non-fermentative characteristics of *E. anophelis*. Lower left: Blood agar showing non-hemolytic, circular, gray-white colonies with smooth edges (1–2 mm in diameter). Lower right: Gram stain of pure colonies from blood agar examined under oil immersion microscopy (1,000 × magnification), revealing Gram-negative bacilli.

Suspected bacterial strains were incubated under aerobic conditions at 35 °C for 48 h. On chocolate agar, colonies were translucent, circular, and gray-white with a glossy surface (1–2 mm in diameter). No growth was observed on MacConkey agar, consistent with the non-fermentative characteristics of *E. anophelis* species. Blood agar supported the development of non-hemolytic, circular, gray-white colonies with smooth edges (1–2 mm in diameter). Pure colonies from blood agar were Gram-stained, revealing Gram-negative bacilli under microscopy (Figure 1).

The six *E. anophelis* strains exhibited a high degree of consistency in their resistance to various antibiotics (Table 2). All strains showed resistance to fluoroquinolones (ciprofloxacin, levofloxacin), *β*-lactams (piperacillin/tazobactam, ticarcillin/clavulanic acid, cefoperazone/sulbactam, ceftazidime, cefpirome), carbapenems (imipenem, meropenem), and aminoglycosides (amikacin, tobramycin). Notably, minocycline was the only agent demonstrating universal susceptibility, with all strains exhibiting MIC values ≤1 μg/mL (6/6, 100% susceptible). For trimethoprim-sulfamethoxazole, five strains (EA1, EA2, EA3, EA4, EA6) were susceptible, while strain EA5 exhibited resistance, resulting in an 83% (5/6) susceptibility rate for this agent.

**Table 2:** Antibiotic resistance testing results for six *E. anophelis* isolates.

Antibiotic class	Antibiotic name	Interpretive categories and MIC breakpoints (μg/mL)			EA1	EA2	EA3	EA4	EA5	EA6
		S	I	R						
Fluoroquinolones	Ciprofloxacin	≤1	2	≥4	≥4 (R)	≥4 (R)	≥4 (R)	≥4 (R)	≥4 (R)	≥4 (R)
	Levofloxacin	≤2	4	8	≥8 (R)	≥8 (R)	≥8 (R)	≥8 (R)	≥8 (R)	≥8 (R)
β-Lactams	Piperacillin/Tazobactam	≤16/4	32/4–64/4	≥128/4	≥128/4 (R)	≥128/4 (R)	≥128/4 (R)	≥128/4 (R)	≥128/4 (R)	≥128/4 (R)
	Ticarcillin/Clavulanate	≤16/2	32/2–64/2	≥128/2	≥128/2 (R)	≥128/2 (R)	≥128/2 (R)	≥128/2 (R)	≥128/2 (R)	≥128/2 (R)
	Cefoperazone/Sulbactam	≤16	32	≥64	≥64 (R)	≥64 (R)	≥64 (R)	≥64 (R)	≥64 (R)	≥64 (R)
	Ceftazidime	≤8	16	≥32	≥64 (R)	≥64 (R)	≥64 (R)	≥64 (R)	≥64 (R)	≥64 (R)
	Cefepime	≤8	16	≥32	≥32 (R)	≥32 (R)	≥32 (R)	≥32 (R)	≥32 (R)	≥32 (R)
Carbapenems	Imipenem	≤4	8	≥16	≥16 (R)	≥16 (R)	≥16 (R)	≥16 (R)	≥16 (R)	≥16 (R)
	Meropenem	≤4	8	≥16	≥16 (R)	≥16 (R)	≥16 (R)	≥16 (R)	≥16 (R)	≥16 (R)
Aminoglycosides	Amikacin	≤16	32	≥64	≥64 (R)	≥64 (R)	≥64 (R)	≥64 (R)	≥64 (R)	≥64 (R)
	Tobramycin	≤4	8	≥16	≥16 (R)	≥16 (R)	≥16 (R)	≥16 (R)	≥16 (R)	≥16 (R)
Tetracyclines	Minocycline	≤4	8	≥16	≤1 (S)	≤1 (S)	≤1 (S)	≤1 (S)	≤1 (S)	≤1 (S)
Sulfonamides	Trimethoprim-Sulfamethoxazole	≤2/38	-	≥4/76	≤2/38 (S)	≤2/38 (S)	≤2/38 (S)	≤2/38 (S)	≥4/76 (R)	≤2/38 (S)

### 3.3 Phylogenetic analysis of pathogen 16S rRNA

**Figure 2:**
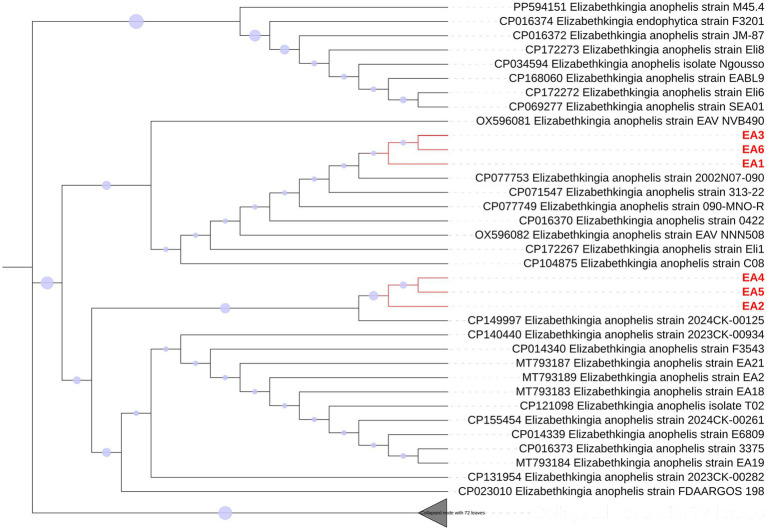
Phylogenetic clustering of six *E. anophelis* isolates based on 16S rRNA. The phylogenetic history was inferred using the Neighbor-Joining method. The tree is drawn to scale as a cladogram (topology tree), where branch lengths are not proportional to evolutionary distances but are optimized for clarity in displaying topological relationships. Blue circles at branch nodes indicate bootstrap support values (1,000 replicates); the area of each circle is proportional to the bootstrap percentage. Reference strains are labeled with their GenBank accession numbers and geographic origins.

Phylogenetic analysis based on 16S rRNA gene sequences grouped the six strains into two clusters (Figure 2). Cluster I (EA1, EA3, EA6) and Cluster II (EA2, EA4, EA5) exhibited a single nucleotide polymorphism (SNP) at position 73 of the coding region: adenine (A) in Cluster I versus guanine (G) in Cluster II. This SNP likely represents a geographic characteristic that distinguishes the two Cluster.

Cluster I shares close homology with the reference strain 0422 (GenBank accession no. CP016370), first isolated from a bloodstream infection in the United States in 1950. Despite its origin in America, this lineage has become the prevalent strain in East and Southeast Asia over the past decade, with recent isolates from Vietnam (accession nos. OX596081, OX596082), Malaysia (accession no. CP172267), and Taiwan (accession nos. CP077753, CP071547, CP104875) suggesting its dominance outbreaks across these regions. In contrast, Cluster II aligns with strain 3,375 (accession no. CP016373), initially identified in the U. S. in 1957. This lineage, formerly prevalent in Europe, North America, and Australia, has recently emerged in mainland China, as evidenced by accessions nos. MT793183, MT793184, MT793187, and MT793189 isolated post-2020. Geographic differences between clusters suggest that there may be potential adaptive evolution driven by regional antimicrobial measures or host-pathogen interactions.

### 3.4 Analysis of pathogen antibiotic resistance genes

**Figure 3:**
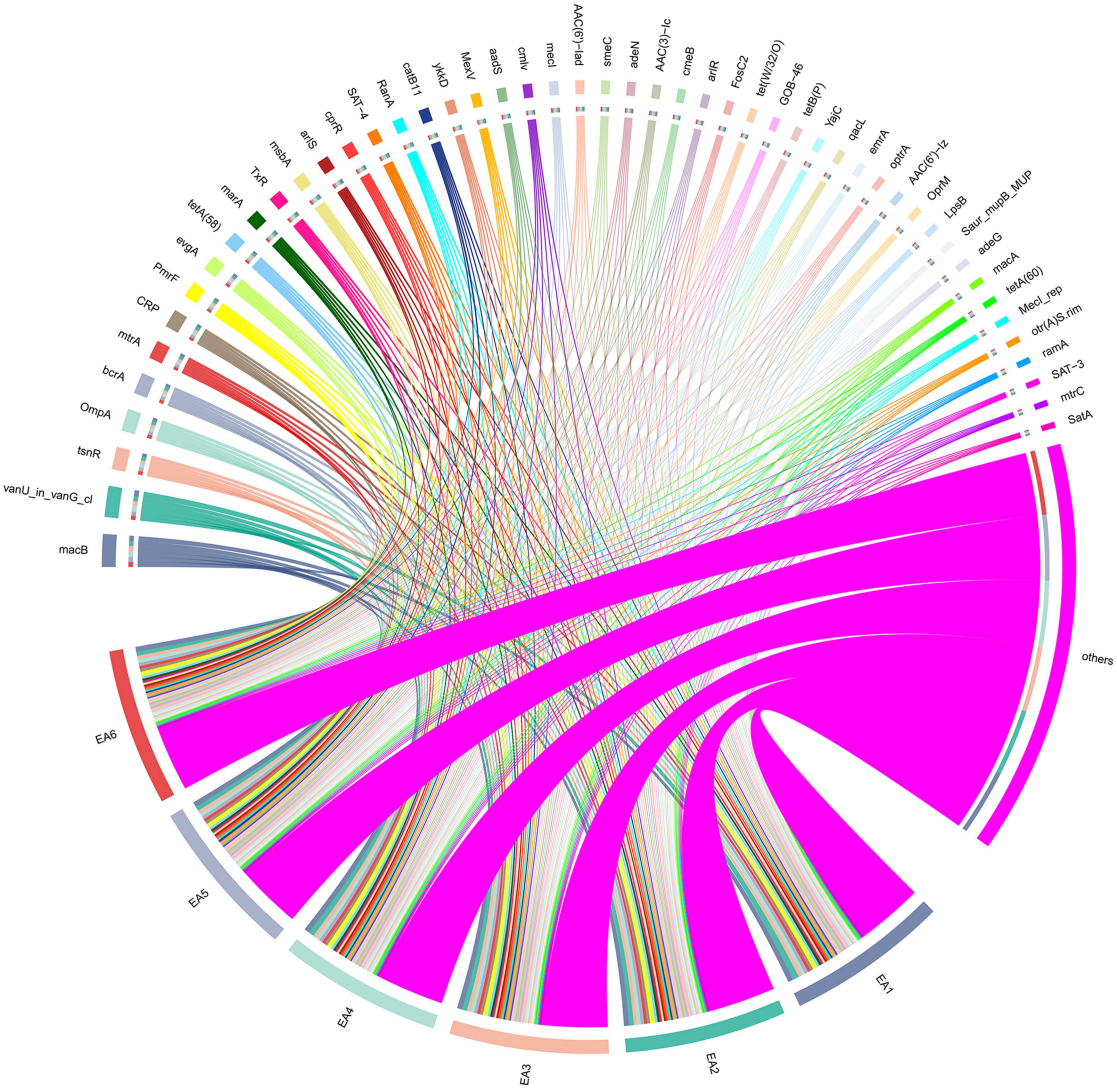
The abundance and association network of antibiotic resistance genes in 6 *E. anophelis* isolates. Chord diagram illustrating the top 50 antibiotic resistance genes (ranked by relative abundance) detected in six clinical strains (EA1–EA6, represented by magenta sectors at bottom). Each ribbon connects a resistance gene (labeled on outer arc) to the strain(s) in which it was detected; ribbon width is proportional to the gene’s relative abundance (TPM-normalized values, see Methods 2.4 Analysis of Resistance and Virulence Genes). Detected resistance genes confer protection against multiple antibiotic classes, including macrolides (e.g., *macB, mtrA*), aminoglycosides [e.g., *AAC(6′)-Iad, aadS*], tetracyclines [e.g., *tetA(58)*], fluoroquinolones (e.g., *evgA, marA*), and glycopeptides (e.g., *vanU_in_vanG_cl*). The strain-specific variations in gene abundance—such as elevated *AAC(6′)-Iad/aadS* and reduced *qacL/OmpA* in EA5—are detailed in Results section 3.4 and Supplementary Table 1.

Comprehensive resistance gene profiling of six *E. anophelis* strains revealed that these strains carry multiple resistance genes (Figure 3; Supplementary Table 1). The identified resistance genes conferred protection against multiple antibiotic classes, including macrolide (e.g., *macB, mtrA, CRP*), peptide (e.g., *OmpA, bcrA, tsnR*), tetracycline (e.g., t*etA(58), TxR, tet(W/32/O)*), aminoglycoside (e.g., *AAC(6′)-Iad, AAC(3)-Ic, aadS*), fluoroquinolone (e.g., *evgA, CRP, marA*), and glycopeptide (e.g., *vanU_in_vanG_cl, YajC*). These strains also shared core resistance mechanisms included antibiotic efflux (e.g., *qacL, tetA(58)*), antibiotic target alteration (e.g., *vanU_in_vanG_cl, PmrF*), antibiotic inactivation (e.g., *AAC(6′)-Iad, aadS*), antibiotic target replacement (e.g., *mecI, dfrA3*), and reduced permeability to antibiotics (e.g., *OmpA, marA*).

Strain-specific resistance gene abundance patterns revealed inter-isolate heterogeneity despite shared core mechanisms: EA1 showed high relative abundance of *vanU_in_vanG_cl* and *bcrA*; EA2 showed high relative abundance of *MecI, SAT-4, evgA, FosC2*, and *CRP*; EA3 and EA4 displayed increased *tsnR* but reduced *qacL* relative abundance; EA5 strain had high relative abundance of *AAC(6′)-Iad, AAC(3)-Ic, aadS, catB11, LnuH*, and *MYO-1*, alongside low *qacL, tetA(58), ykkD, evgA, OmpA,* and *marA*; EA6 showed high relative abundance of *mecI* and *FosC2*.

### 3.5 Analysis of pathogen virulence genes

**Figure 4:**
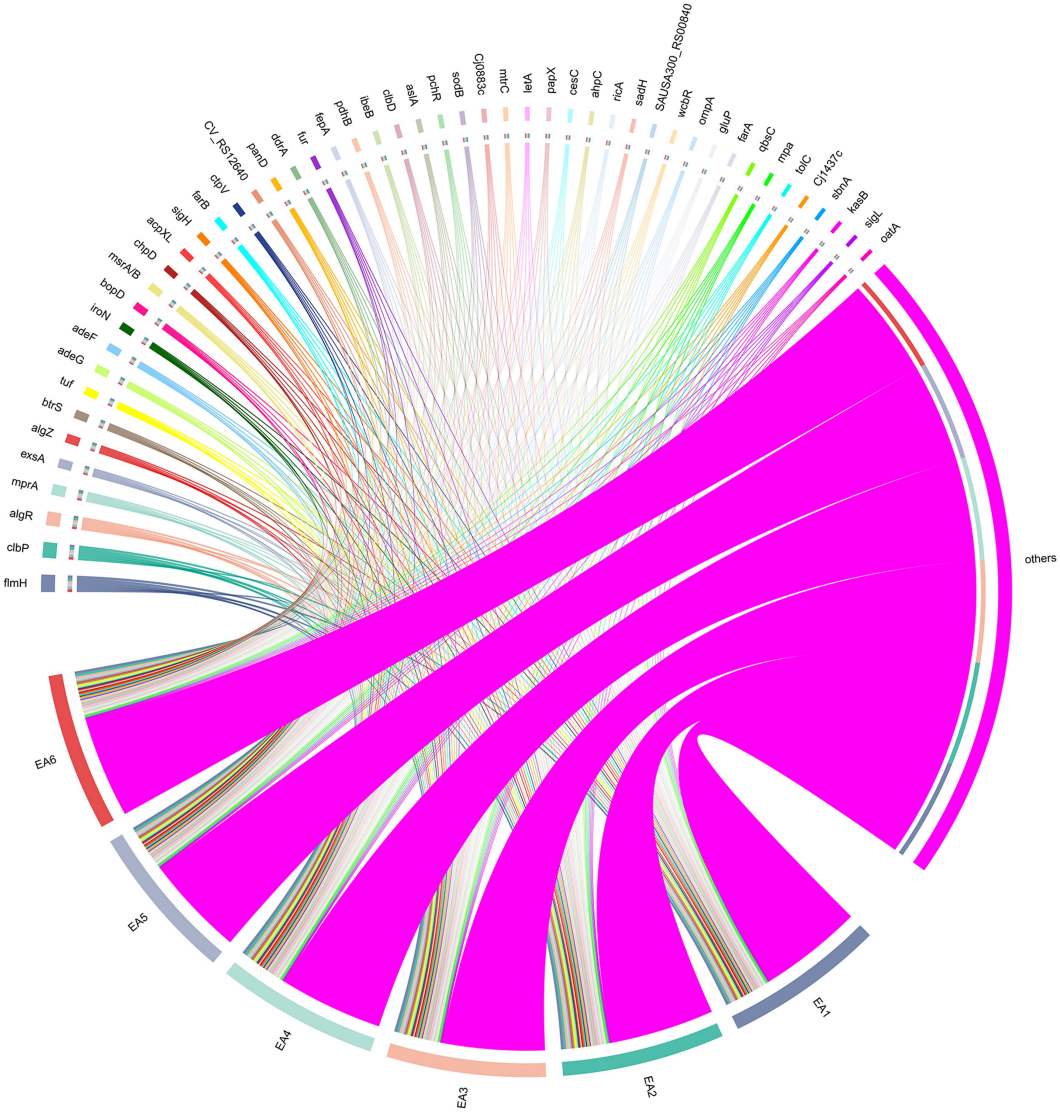
The abundance and association network of virulence genes in 6 *E. anophelis* isolates. Chord diagram depicting the top 50 virulence genes (ranked by relative abundance) detected in six clinical strains (EA1–EA6, represented by magenta sectors at bottom). Each ribbon connects a virulence gene (labeled on outer arc) to the strain(s) in which it was detected; ribbon width is proportional to the gene’s relative abundance (TPM-normalized values, see Methods 2.4 analysis of resistance and virulence genes). Detected virulence genes are associated with bacterial motility (e.g., *flmH*), exotoxin production (e.g., *clbP, clbD*), biofilm formation (e.g., *algR, algZ*), immune regulation (e.g., *mprA, sigH*), and effector delivery systems (e.g., *exsA, btrS*). The dense connectivity pattern reflects conserved core pathogenicity determinants across all strains, while strain-specific variations—notably EA5’s unique genetic signature (absence of *cesH*, presence of *sigE*, elevated *pagR-XO2*, reduced *sodB/ddrA*)—are detailed in Results section 3.5 and Supplementary Table 2.

Virulence gene analysis of the six *E. anophelis* strains revealed conserved core pathogenicity determinants (Figure 4; Supplementary Table 2). Genes with high abundance included those associated with bacterial movement (e.g., *flmH*), exotoxin production (e.g., *clbP, clbD*), biofilm formation (e.g., *algR, algZ*), immune regulation (e.g., *mprA, sigH*), and effector delivery systems (e.g., *exsA, btrS*).

Although the core virulence mechanisms are consistently present across all strains, distinct genomic signatures were observed: EA5 exhibited a unique genetic signature, lacking the *cesH* gene while uniquely carrying the *sigE* gene. This strain showed elevated relative abundance of *pagR-XO2*, *SAUSA300_RS00840, ROD_RS25695, ML_RS08565*, and *fotS*, alongside reduced *PMI_RS02630, hopJ1, mprA, sodB,* and *ddrA*. EA1 showed reduced relative abundance of *PMI_RS02630, ML_RS08565, ahpC,* and *M3Q_RS01450*; EA2 showed elevated *PMI_RS02630, algR, hopJ1, panD, ahpC, btrS, pchR,* and *msrA/B* relative abundance; EA3 displayed high relative abundance of *mprA* and *pagR*-*XO1*. EA4 had high relative abundance of *acpXL*, *adeG*, *fur*, and *clbP*. EA6 exhibited high relative abundance of *acpXL*, *PMI_RS02630, bopD, btrS,* and *papX*, contrasting with low relative abundance of *ROD_RS25695, ML_RS08565, Cj0883c, adeG, panD, adeF,* and *ddrA*.

## 4 Discussion

In this study, six *E. anophelis* strains were isolated from clinical specimens of hospitalized patients. Phylogenetic analysis based on the 16S rRNA gene revealed two distinct clusters. Cluster I strains (EA1, EA3, EA6) carried adenine (A) at position 73 of the 16S rRNA gene and demonstrated high phylogenetic similarity with isolates prevalent in East and Southeast Asia, while Cluster II (EA2, EA4, EA5) strains harbored guanine (G) at this locus and aligned closely with strains from Europe, North America, and Australia.

In recent years, infections caused by *Elizabethkingia* species have increased significantly worldwide (Zajmi et al., 2022), with *E. anophelis* emerging as a particularly concerning pathogen due to its intrinsic multidrug resistance and high mortality rates in immunocompromised patients, posing a serious public health threat (Hu et al., 2022b). Current diagnostic challenges stem from overlapping clinical presentations and biochemical similarities between *E. anophelis* and *E. meningoseptica*, and this diagnostic ambiguity may lead to a systemic underestimation of the true epidemiological burden (Spencer et al., 2020). Our phylogenetic data suggest transmission of these lineages since the mid-20th century (Lin et al., 2022), with modern population mobility posing risks for cross-border spread.

To address these diagnostic and surveillance challenges, we recommend healthcare institutions to regularly update microbial identification platforms (e.g., VITEK® 2 and MALDI-TOF MS) to improve species-level resolution for *Elizabethkingia* (Cheng et al., 2019), and establish molecular surveillance networks to monitor strain distribution patterns, detect emerging lineages, and support infection control interventions (McTaggart et al., 2019; Li et al., 2021).

The six *E.anophelis* strains investigated in this study exhibited multidrug resistance to *β*-lactams, carbapenems, aminoglycosides, and fluoroquinolones. Genomic analysis via the CARD confirmed the prevalence of five core resistance mechanisms: antibiotic efflux, antibiotic target alteration, antibiotic inactivation, antibiotic target replacement and reduced permeability to antibiotics. Notably, strain EA5 displayed unique genomic signatures: elevated relative abundance of *AAC (6′)-Iad, AAC (3)-Ic,* and *aadS* (aminoglycoside inactivation), alongside high *catB11* and *LnuH* abundance (chloramphenicol/lincosamide resistance) (Luo et al., 2018; Ghafoori et al., 2021). Concurrently, reduced *OmpA* gene relative abundance in EA5 may impair outer membrane permeability (Chiang et al., 2022), synergizing with *marA*-regulated efflux pump activation to further diminish intracellular antibiotic accumulation, thereby potentially contributing to its distinct resistance profile (Darby et al., 2023).

Despite the genomic detection of tetracycline resistance genes (*tetA(58), TxR, tet(W/32/O)*), all strains retained phenotypic susceptibility to minocycline in broth microdilution assays. This discrepancy between genotypic prediction and phenotypic drug sensitivity emphasizes the multifactorial regulation of resistance gene expression, and thus the need to combine antimicrobial susceptibility testing with genomic data for clinical decision-making. Our findings align with prior studies reporting 60–100% minocycline susceptibility in *Elizabethkingia* spp. (Lin et al., 2018), as its enhanced lipophilicity enables superior penetration of bacterial outer membranes and reduced susceptibility to efflux compared to other tetracyclines, potentially explaining its retained activity despite resistance gene carriage.

Virulence gene profiling confirmed that all six *E. anophelis* strains harbored critical virulence determinants associated with bacterial movement, exotoxin production, biofilm formation, immune regulation, and effector delivery systems. Elevated abundance of biofilm-related genes (*algR/algZ*) may enhance bacterial adherence to medical devices (e.g., catheters), aligning with biofilm formation to nosocomial outbreaks (Hu et al., 2022a; Sánchez-Jiménez et al., 2023). The activation of exotoxin genes *clbP/clbD* may induce DNA interstrand crosslinking, exacerbating host tissue damage (Adnani et al., 2017).

Strain EA5 exhibited a distinctive virulence gene expression profile compared to other strains (Supplementary Table 2). Notably, EA5 completely lacked expression of *CT_473* (effector delivery system) and *cesH* (exotoxin), while uniquely expressing *sigE*, a gene encoding an alternative sigma factor that was absent in all other strains. Alternative sigma factors are known to regulate stress response pathways and virulence gene expression in diverse bacterial pathogens (Kazmierczak et al., 2005; Hengge, 2008), though their specific roles in *Elizabethkingia* remain to be characterized. Additionally, EA5 displayed elevated expression of regulatory genes (*pagR-XO2*: TPM = 587.35 vs. mean 375.10) and adherence-related genes (*ROD_RS25695, ML_RS08565*), alongside reduced expression of *sodB* (superoxide dismutase) and *ddrA* (immune modulation). Reduced *sodB* expression may compromise defense against oxidative stress, a critical factor in host-pathogen interactions (Imlay, 2013). These strain-specific variations underscore the importance of genome-level profiling to capture inter-strain heterogeneity that is not evident from phenotypic antimicrobial susceptibility testing alone.

The intrinsic multidrug resistance observed across all isolates—including both community-acquired and nosocomial strains—suggests this resistance mechanisms represent native properties of *E. anophelis* (Breurec et al., 2016; Andriyanov et al., 2024). Despite this shared resistance foundation, the observed inter-strain heterogeneity in resistance and virulence gene abundance reflects diverse evolutionary pressures acting on *E. anophelis* across different ecological niches. For the predominant community-acquired infections in this cohort (5/6 cases), evolutionary drivers likely include environmental antimicrobials in water distribution systems, biofilm formation, and interactions with environmental hosts (Breurec et al., 2016). In contrast, the healthcare-associated strain (EA4) may experience direct selective pressure from prolonged antibiotic exposure within clinical settings. These findings necessitate integrated genomic surveillance encompassing clinical and environmental sources to comprehensively track *E. anophelis* transmission dynamics and evolutionary trajectories.

All enrolled patients presented with multiple comorbidities (e.g., hepatobiliary diseases, malignancies). The severity of underlying conditions, prolonged hospitalization, and extensive antibiotic exposure contributed to complicated infection management (Lau et al., 2016; Seong et al., 2020). It is important to note that half of the infections (3/6) were polymicrobial; however, *E. anophelis* was isolated from sterile sites (blood or bile) in 6 enrolled patients, confirming its invasive pathogenic role. For patients with confirmed multidrug-resistant *E. anophelis* infections, treatment strategies should be guided by antimicrobial susceptibility testing. Based on our findings, combination therapy should focus on effective agents such as minocycline-based regimens, with treatment selection individualized based on susceptibility profiles and patient factors (Spencer et al., 2020). Source control measures (e.g., catheter removal, biliary drainage) are essential adjuncts to antimicrobial therapy in device-associated or obstructive infections.

This study has the following limitations: (1) the single-center study design with a small sample size (*n* = 6) limits generalizability of findings; (2) antibiotic exposure history depended on electronic records, which may not capture undocumented treatments (e.g., prior outpatient antibiotic use or self-medication); (3) resistance gene analysis (e.g., *aac(6′)-Iad*) and virulence gene detection (e.g., *sigE*) were based on whole-genome sequencing data, requiring functional validation to confirm gene expression and activity; (4) retrospective design limited our ability to trace infection sources for community-acquired cases, and prior healthcare exposures cannot be definitively excluded.

## 5 Conclusion

This study characterizes six *E. anophelis* strains from Shanghai hospitalized patients, revealing critical insights into this pathogen’s antimicrobial resistance, virulence, and phylogenetic diversity. Phylogenetic analysis identified two distinct clusters: Cluster I aligning with East/Southeast Asian isolates, and Cluster II showing affinity with strains from diverse origins, suggesting independent introduction events and cross-border transmission potential. All strains exhibited extensively drug-resistant phenotypes with five core mechanisms: antibiotic efflux, antibiotic target alteration, antibiotic inactivation, antibiotic target replacement, and reduced permeability to antibiotics. Virulence profiling revealed pathogenicity determinants for bacterial movement, exotoxin production, biofilm formation, immune regulation, and effector delivery systems. Despite tetracycline resistance genes, all strains retained minocycline susceptibility, supporting its therapeutic value. Strain EA5 displayed unique genomic signatures with distinct resistance and virulence gene profiles, underscoring the necessity of genome-level profiling beyond phenotypic antimicrobial susceptibility testing. These findings advocate for: (1) enhanced diagnostics through updated identification platform; (2) minocycline-based regimens guided by antimicrobial susceptibility testing; (3) regional molecular surveillance networks to monitor strain evolution and transmission; (4) larger studies to validate genotype–phenotype correlations using transcriptomic/proteomic approaches and high-resolution typing.

## Data Availability

The datasets presented in this study can be found in online repositories. The names of the repository/repositories and accession number(s) can be found at: https://www.ncbi.nlm.nih.gov/genbank/, PV715948, PV715949, PV715950, PV715951, PV715952, and PV715953.

## Supplementary material

SUPPLEMENTARY Table 1 Gene Abundance (TPM) of antibiotic resistance genes across six clinical Elizabethkingia anophelis isolates.

SUPPLEMENTARY Table 2 Gene Abundance (TPM) of virulence genes across six clinical Elizabethkingia anophelis isolates.
