# Supplementary material for: Genomic profiling of Elizabethkingia anophelis clinical isolates from a Shanghai hospital: phylogenetic divergence coexists with heterogeneous antibiotic resistance and virulence determinants
Source: Front Microbiol. 2026 Jan 16;16:1751256. doi: 10.3389/fmicb.2025.1751256 (PMC12855470; doi:10.3389/fmicb.2025.1751256)
Supplement: SUPPLEMENTARY Table 1 — Gene Abundance (TPM) of antibiotic resistance genes across six clinical Elizabethkingia anophelis isolates. [file Table_1.docx]

**Supplementary Table 1. Gene Abundance (TPM) of antibiotic resistance genes of six clinical Elizabethkingia anophelis isolates**

| **Gene Symbol** | **Resistance Mechanism** | **EA1 (TPM)** | **EA2 (TPM)** | **EA3 (TPM)** | **EA4 (TPM)** | **EA5 (TPM)** | **EA6 (TPM)** |
| --- | --- | --- | --- | --- | --- | --- | --- |
| vanU_in_vanG_cl | antibiotic target alteration | 2006.2 | 1539.53 | 1577.59 | 1767.75 | 1403.76 | 1329.85 |
| AAC(6')-Iad | antibiotic inactivation | 432.292 | 392.306 | 390.67 | 420.597 | 748.973 | 422.761 |
| AAC(3)-Ic | antibiotic inactivation | 412.65 | 369.603 | 377.801 | 375.051 | 679.575 | 417.038 |
| aadS | antibiotic inactivation | 446.716 | 441.476 | 435.25 | 460.605 | 669.305 | 448.429 |
| catB11 | antibiotic inactivation | 522.991 | 510.717 | 515.546 | 495.764 | 706.478 | 514.271 |
| LnuH | antibiotic inactivation | 241.414 | 219.186 | 219.788 | 224.431 | 417.814 | 229.425 |
| MYO-1 | antibiotic inactivation | 233.701 | 241.924 | 228.296 | 224.919 | 395.337 | 204.829 |
| qacL | antibiotic efflux | 401.395 | 415.28 | 355.134 | 293.547 | 297.689 | 453.291 |
| mecI | antibiotic target replacement | 425.744 | 516.338 | 448.48 | 543.313 | 438.863 | 522.243 |
| tetA(58) | antibiotic efflux | 730.091 | 768.682 | 804 | 787.008 | 684.422 | 711.745 |
| ykkD | antibiotic efflux | 474.362 | 491.871 | 538.522 | 563.668 | 445.878 | 501.876 |
| SAT-4 | antibiotic inactivation | 541.858 | 629.548 | 574.365 | 561.553 | 530.544 | 560.925 |
| evgA | antibiotic efflux | 798.925 | 850.503 | 792.752 | 778.827 | 754.879 | 822.883 |
| FosC2 | antibiotic inactivation | 384.97 | 441.855 | 377.979 | 387.521 | 367.412 | 442.988 |
| CRP | antibiotic efflux | 918.017 | 1001.11 | 945.536 | 968.667 | 916.226 | 948.717 |
| macB | antibiotic efflux | 1714.66 | 1659.72 | 1699.23 | 1737.72 | 1679.55 | 1656.89 |
| OmpA | reduced permeability to antibiotic | 1174.8 | 1163.34 | 1142.16 | 1173.91 | 1091.56 | 1140.57 |
| bcrA | antibiotic efflux | 1126.17 | 1094.23 | 1064.44 | 1129 | 1086.46 | 1059.75 |
| marA | antibiotic efflux; reduced permeability to antibiotic | 755.934 | 732.382 | 714.106 | 724.623 | 679.636 | 708.709 |
| tsnR | antibiotic target alteration | 1139.42 | 1139.8 | 1187.28 | 1187.49 | 1146.69 | 1179.35 |
| PmrF | antibiotic target alteration | 840.656 | 845.744 | 823.027 | 877.894 | 836.349 | 808.034 |
| RanA | antibiotic efflux | 590.946 | 551.216 | 566.32 | 585.362 | 559.049 | 527.805 |
| mgrA | antibiotic efflux | 284.834 | 260.06 | 256.125 | 287.913 | 254.714 | 312.497 |
| mtrA | antibiotic efflux | 969.915 | 945.025 | 974.746 | 969.463 | 922.168 | 929.909 |
| dfrA3 | antibiotic target replacement | 258.464 | 251.582 | 275.065 | 262.069 | 210.449 | 261.633 |
| SatA | antibiotic inactivation | 288.423 | 295.461 | 329.835 | 339.906 | 303.639 | 289.594 |
| cprR | antibiotic target alteration; antibiotic efflux | 573.618 | 573.1 | 608.853 | 613.351 | 564.499 | 574.132 |
| arlR | antibiotic efflux | 408.744 | 421.878 | 422.852 | 386.406 | 377.662 | 427.01 |
| MexV | antibiotic efflux | 505.391 | 496.123 | 475.002 | 497.847 | 482.418 | 448.565 |
| QnrB15 | antibiotic target protection | 241.297 | 259.204 | 241.298 | 250.311 | 199.658 | 238.33 |
| TxR | antibiotic efflux | 668.131 | 677.944 | 635.888 | 697.083 | 661.168 | 677.008 |
| vatB | antibiotic inactivation | 311.186 | 308.499 | 313.587 | 268.823 | 278.622 | 304.046 |
| YajC | antibiotic efflux | 395.279 | 353.922 | 378.797 | 378.673 | 404.742 | 365.631 |
| SAT-3 | antibiotic inactivation | 304.633 | 307.461 | 341.39 | 292.57 | 325.483 | 331.901 |
| arlS | antibiotic efflux | 644.397 | 602.046 | 622.806 | 622.523 | 596.518 | 608.486 |
| MecI_rep | antibiotic target replacement | 301.762 | 335.278 | 314.086 | 317.828 | 346.518 | 340.09 |
| ramA | antibiotic efflux; reduced permeability to antibiotic | 300.861 | 336.592 | 322.047 | 339.982 | 300.977 | 316.435 |
| AAC(6')-Iz | antibiotic inactivation | 387.035 | 364.852 | 366.712 | 350.975 | 341.85 | 352.463 |
| vanR_in_vanA_cl | antibiotic target alteration | 172.315 | 187.987 | 210.409 | 178.316 | 174.937 | 168.359 |
| cmeB | antibiotic efflux | 420.213 | 420.354 | 404.838 | 426.517 | 401.186 | 386.268 |
| vanW_in_vanG_cl | antibiotic target alteration | 236.958 | 235.901 | 224.043 | 251.007 | 207.364 | 240.035 |
| emrA | antibiotic efflux | 365.259 | 381.46 | 345.88 | 378.166 | 367.594 | 347.785 |
| patA | antibiotic efflux | 274.801 | 271.652 | 260.399 | 303.682 | 275.387 | 269.159 |
| smeC | antibiotic efflux | 462.469 | 476.176 | 439.214 | 472.265 | 453.814 | 446.087 |
| GOB-46 | antibiotic inactivation | 402.129 | 391.105 | 363.363 | 395.293 | 389.77 | 373.619 |
| macA | antibiotic efflux | 328.846 | 322.929 | 344.084 | 336.924 | 335.556 | 302.972 |
| otr(A)S.rim | antibiotic target protection | 315.12 | 341.083 | 305.232 | 335.662 | 327.387 | 310.761 |
| OprZ | antibiotic efflux | 279.075 | 284.073 | 274.177 | 282.001 | 266.903 | 245.454 |
| tetB(P) | antibiotic target protection | 378.161 | 390.156 | 361.399 | 399.784 | 375.523 | 395.021 |
| sta | antibiotic inactivation | 243.578 | 264.693 | 282.939 | 273.454 | 272.457 | 259.112 |
| cmlv | antibiotic inactivation | 505.369 | 484.193 | 468.031 | 489.752 | 479.897 | 470.706 |
| BJP-1 | antibiotic inactivation | 217.772 | 225.514 | 243.993 | 243.473 | 249.53 | 224.261 |
| sul4 | antibiotic target replacement | 159.574 | 176.397 | 175.098 | 149.101 | 181.836 | 178.734 |
| gadW | antibiotic efflux | 208.78 | 189.608 | 181.863 | 191.864 | 192.213 | 215.833 |
| CME-1 | antibiotic inactivation | 172.492 | 176.968 | 154.791 | 149.201 | 156.433 | 146.444 |
| tet(W/32/O) | antibiotic target protection | 384.619 | 381.152 | 377.522 | 403.634 | 406.073 | 381.606 |
| adeN | antibiotic efflux | 465.164 | 440.43 | 460.817 | 450.089 | 433.012 | 442.526 |
| gadX | antibiotic efflux | 170.126 | 183.537 | 172.072 | 167.406 | 155.094 | 148.965 |
| cmeC | antibiotic efflux | 151.255 | 173.579 | 140.959 | 164.404 | 144.926 | 156.833 |
| vanE | antibiotic target alteration | 214.941 | 189.815 | 193.651 | 206.542 | 182.707 | 188.405 |
| adeG | antibiotic efflux | 338.348 | 326.424 | 341.524 | 340.804 | 318.449 | 313.308 |
| mtrC | antibiotic efflux | 321.999 | 320.728 | 333.424 | 305.889 | 300.141 | 311.605 |
| AAC(6')-IIc | antibiotic inactivation | 213.129 | 202.022 | 230.67 | 221.369 | 203.439 | 202.927 |
| farB | antibiotic efflux | 198.992 | 188.412 | 171.976 | 199.606 | 204.747 | 192.168 |
| evgS | antibiotic efflux | 305.823 | 314.093 | 285.24 | 313.437 | 296.162 | 294.869 |
| OpmH | antibiotic efflux | 153.174 | 163.334 | 160.02 | 176.018 | 153.017 | 142.332 |
| vanR_in_vanI_cl | antibiotic target alteration | 229.37 | 233.75 | 226.67 | 202.829 | 219.728 | 213.852 |
| bacA | antibiotic target alteration | 138.98 | 161.617 | 147.796 | 170.19 | 155.757 | 146.936 |
| MSI-1 | antibiotic inactivation | 185.429 | 161.996 | 170.601 | 185.783 | 164.064 | 163.875 |
| MexJ | antibiotic efflux | 142.856 | 154.194 | 135.28 | 143.051 | 124.11 | 129.752 |
| msbA | antibiotic efflux | 664.21 | 667.852 | 645.531 | 661.681 | 641.753 | 656.569 |
| adeJ | antibiotic efflux | 166.298 | 183.569 | 156.748 | 179.081 | 165.137 | 162.829 |
| vanS_in_vanC_cl | antibiotic target alteration | 150.863 | 146.407 | 146.113 | 136.263 | 126.392 | 128.447 |
| almE | antibiotic target alteration | 283.006 | 294.198 | 272.696 | 266.212 | 274.172 | 284.898 |
| efpA | antibiotic efflux | 157.25 | 157.892 | 152.186 | 154.387 | 131.347 | 146.968 |
| Saur_mupB_MUP | antibiotic target alteration | 335.217 | 342.421 | 319.319 | 345.317 | 334.791 | 325.39 |
| oleC | antibiotic efflux | 184.133 | 197.496 | 192.261 | 204.892 | 178.787 | 199.37 |
| CAR-1 | antibiotic inactivation | 169.458 | 157.557 | 185.464 | 180.517 | 169.998 | 172.284 |
| ceoB | antibiotic efflux | 298.518 | 298.02 | 299.899 | 308.531 | 287.99 | 281.403 |
| bcr-1 | antibiotic efflux | 285.231 | 284.401 | 278.657 | 292.637 | 279.3 | 264.373 |
| ALI-1 | antibiotic inactivation | 187.362 | 186.728 | 165.294 | 176.067 | 191.113 | 182.969 |
| RanB | antibiotic efflux | 241.549 | 233.458 | 221.458 | 247.79 | 242.569 | 242.569 |
| arnA | antibiotic target alteration | 192.517 | 176.262 | 187.326 | 194.335 | 171.706 | 177.422 |
| LpsB | reduced permeability to antibiotic | 350.916 | 350.284 | 334.629 | 340.078 | 328.307 | 332.93 |
| Kpne_KpnH | antibiotic efflux | 159.472 | 160.155 | 134.878 | 154.541 | 150.044 | 148.532 |
| Paer_CpxR | antibiotic efflux | 172.087 | 176.123 | 181.892 | 165.032 | 155.049 | 168.17 |
| tet(X1) | antibiotic inactivation | 151.924 | 174.213 | 164.997 | 171.633 | 152.902 | 163.728 |
| vanR_in_vanC_cl | antibiotic target alteration | 192.588 | 198.387 | 195.661 | 184.789 | 173.505 | 185.005 |
| PAM-3 | antibiotic inactivation | 154.583 | 143.733 | 152.037 | 157.818 | 145.492 | 132.827 |
| Rsph_ampC_BLA | antibiotic inactivation | 163.629 | 152.245 | 162.979 | 178.133 | 166.321 | 156.235 |
| MexW | antibiotic efflux | 285.918 | 275.447 | 266.268 | 274.337 | 262.153 | 264.459 |
| SST-1 | antibiotic inactivation | 166.055 | 155.948 | 152.879 | 159.944 | 177.228 | 158.522 |
| adeS | antibiotic efflux | 162.376 | 173.368 | 150.24 | 166.285 | 155.548 | 153.45 |
| cpxA | antibiotic efflux | 303.741 | 317.801 | 299.452 | 292.116 | 306.094 | 309.044 |
| vanH_in_vanO_cl | antibiotic target alteration | 145.979 | 150.272 | 161.653 | 137.231 | 148.008 | 139.568 |
| mdtC | antibiotic efflux | 143.635 | 145.327 | 139.013 | 156.889 | 131.147 | 137.946 |
| pgpB | antibiotic target alteration | 196.756 | 181.678 | 206.296 | 195.905 | 198.267 | 188.508 |
| cmx | antibiotic efflux | 146.83 | 145.972 | 158.342 | 139.782 | 145.407 | 132.751 |
| OprM | antibiotic efflux | 346.977 | 337.123 | 349.296 | 360.541 | 358.356 | 352.667 |
| BlaB-1 | antibiotic inactivation | 200.65 | 194.706 | 200.012 | 182.181 | 186.948 | 182.361 |
| CSA-1 | antibiotic inactivation | 194.101 | 205.57 | 201.283 | 216.358 | 214.091 | 201.397 |
| tet(35) | antibiotic efflux | 165.665 | 147.882 | 158.373 | 151.658 | 146.656 | 143.244 |
| vanR_in_vanF_cl | antibiotic target alteration | 202.883 | 214.093 | 200.266 | 193.176 | 191.695 | 194.865 |
| MexF | antibiotic efflux | 146.137 | 154.23 | 148.967 | 165.891 | 149.551 | 141.862 |
| vanT_in_vanG_cl | antibiotic target alteration | 175.482 | 190.264 | 165.481 | 173.745 | 173.596 | 180.517 |
| ACC-1d | antibiotic inactivation | 158.458 | 158.135 | 168.634 | 174.552 | 153.907 | 170.268 |
| bcrC | antibiotic target alteration | 156.811 | 148.862 | 150.941 | 137.135 | 160.65 | 148.211 |
| MOX-6 | antibiotic inactivation | 143.526 | 166.334 | 148.738 | 153.41 | 146.639 | 151.581 |
| adeC | antibiotic efflux | 157.92 | 164.063 | 149.453 | 155.743 | 152.523 | 140.718 |
| Ecol_acrA | antibiotic efflux | 203.857 | 198.508 | 196.764 | 218.644 | 208.908 | 205.245 |
| OCH-6 | antibiotic inactivation | 155.045 | 167.227 | 152.472 | 161.612 | 144.406 | 154.487 |
| tetA(60) | antibiotic efflux | 333.212 | 340.077 | 319.61 | 321.173 | 325.387 | 329.169 |
| tet(48) | antibiotic inactivation | 169.776 | 173.935 | 158.152 | 162.925 | 157.233 | 154.384 |
| MexA | antibiotic efflux | 136.969 | 147.681 | 149.375 | 139.875 | 137.586 | 128.426 |
| kdpD | antibiotic efflux | 166.167 | 169.791 | 155.637 | 171.251 | 156.17 | 173.049 |
| otr(B) | antibiotic efflux | 178.472 | 177.625 | 171.106 | 188.639 | 178.474 | 191.461 |
| tet(V) | antibiotic efflux | 181.686 | 171.435 | 178.899 | 169.977 | 166.61 | 186.095 |
| CMY-113 | antibiotic inactivation | 154.195 | 153.203 | 147.659 | 148.189 | 133.283 | 144.26 |
| clbC | antibiotic target alteration | 164.977 | 152.943 | 162.019 | 173.625 | 155.241 | 158.178 |
| sdiA | antibiotic efflux | 158.595 | 142.451 | 164.561 | 152.985 | 150.8 | 152.266 |
| facT | antibiotic efflux | 171.332 | 175.07 | 167.568 | 159.488 | 178.854 | 162.348 |
| mef(C) | antibiotic efflux | 135.929 | 139.929 | 143.34 | 138.412 | 130.353 | 122.83 |
| mecA | antibiotic target replacement | 178.873 | 189.73 | 173.843 | 188.162 | 179.585 | 192.51 |
| ACT-55 | antibiotic inactivation | 159.85 | 171.036 | 160.827 | 150.014 | 163.881 | 155.011 |
| SPU-1 | antibiotic inactivation | 167.004 | 169.67 | 167.42 | 170.276 | 163.249 | 150.905 |
| mdtH | antibiotic efflux | 130.785 | 146.725 | 133.737 | 138.087 | 127.475 | 128.731 |
| BahA | antibiotic inactivation | 179.42 | 159.86 | 166.534 | 170.067 | 163.498 | 162.333 |
| NmcR | antibiotic inactivation | 146.765 | 163.77 | 152.096 | 155.404 | 152.262 | 164.098 |
| optrA | antibiotic target protection | 356.324 | 366.624 | 354.101 | 372.107 | 366.382 | 359.861 |
| farA | antibiotic efflux | 158.986 | 157.533 | 152.375 | 163.833 | 172.069 | 164.339 |
| mexM | antibiotic efflux | 195.782 | 178.162 | 181.766 | 188.004 | 179.333 | 185.174 |
| EC-8 | antibiotic inactivation | 186.878 | 188.288 | 180.007 | 198.432 | 185.5 | 193.579 |
| MCR-10.4 | antibiotic target alteration | 127.144 | 137.351 | 139.672 | 145.582 | 137.85 | 131.672 |
| mdsB | antibiotic efflux | 131.452 | 136.82 | 133.755 | 134.981 | 125.59 | 120.529 |
| DHA-29 | antibiotic inactivation | 147.886 | 144.419 | 147.449 | 143.827 | 139.616 | 131.315 |
| abeM | antibiotic efflux | 134.913 | 138.757 | 127.427 | 137.611 | 132.601 | 123.099 |
| Saur_mupA_MUP | antibiotic target alteration | 178.487 | 178.734 | 169.315 | 188.512 | 178.308 | 179.426 |
| mecB | antibiotic target replacement | 186.26 | 169.263 | 172.07 | 179.156 | 178.607 | 177.916 |
| AxyY | antibiotic efflux | 137.811 | 141.579 | 133.669 | 140.5 | 132.13 | 126.041 |
| MexH | antibiotic efflux | 146.583 | 163.457 | 153.48 | 157.188 | 150.339 | 152.268 |
| vanS_in_vanL_cl | antibiotic target alteration | 174.819 | 181.01 | 163.856 | 174.607 | 169.599 | 171.51 |
| tet(45) | antibiotic efflux | 160.723 | 164.124 | 152.25 | 162.111 | 151.581 | 152.237 |
| tet(41) | antibiotic efflux | 146.861 | 146.264 | 136.566 | 141.475 | 134.452 | 147.09 |
| cdeA | antibiotic efflux | 153.105 | 139.89 | 142.676 | 148.07 | 139.503 | 140.27 |
| PNGM-1 | antibiotic inactivation | 144.836 | 151.865 | 150.458 | 153.186 | 160.597 | 147.736 |
| smeS | antibiotic efflux | 162.641 | 163.807 | 164.43 | 169.046 | 161.108 | 152.769 |
| GOB-20 | antibiotic inactivation | 176.216 | 188.149 | 179.711 | 184.215 | 179.296 | 189.74 |
| EreD | antibiotic inactivation | 133.58 | 140.456 | 145.014 | 136.27 | 139.553 | 130.211 |
| tet(44) | antibiotic target protection | 139.379 | 142.404 | 144.72 | 140.804 | 144.161 | 130.388 |
| mdtN | antibiotic efflux | 157.185 | 165.934 | 159.827 | 171.853 | 160.487 | 162.108 |
| vanH_in_vanB_cl | antibiotic target alteration | 144.766 | 145.554 | 144.838 | 150.975 | 136.474 | 138.814 |
| vanR_in_vanE_cl | antibiotic target alteration | 149.751 | 152.806 | 148.296 | 140.557 | 153.329 | 145.263 |
| vanH_in_vanA_cl | antibiotic target alteration | 209.317 | 213.04 | 213.169 | 222.668 | 218.412 | 212.876 |
| TolC | antibiotic efflux | 188.498 | 194.763 | 194.548 | 194.991 | 201.781 | 200.361 |
| Erm(30) | antibiotic target alteration | 156.772 | 159.701 | 160.43 | 170.348 | 164.927 | 163.013 |
| Saur_LmrS | antibiotic efflux | 144.674 | 148.025 | 156.245 | 148.145 | 146.446 | 142.585 |
| rosB | antibiotic efflux | 299.225 | 304.92 | 293.552 | 297.59 | 295.892 | 291.681 |
| Ctra_murA_FOF | antibiotic target alteration | 177.814 | 177.21 | 174.352 | 186.858 | 179.452 | 175.921 |
| Abau_AmvA | antibiotic efflux | 141.874 | 143.204 | 147.694 | 144.644 | 136.888 | 138.051 |
| mreA | antibiotic efflux | 163.86 | 157.026 | 166.95 | 166.199 | 159.915 | 159.233 |
| novA | antibiotic efflux | 117.949 | 120.962 | 116.288 | 123.086 | 118.009 | 111.88 |
| CMY-82 | #N/A | 154.505 | 157.368 | 147.449 | 158.368 | 154.748 | 154.783 |
| TaeA | antibiotic efflux | 163.019 | 160.303 | 165.732 | 169.735 | 162.89 | 167.691 |
| adeK | antibiotic efflux | 135.341 | 130.581 | 132.198 | 125.781 | 127.695 | 128.936 |
| BUT-2 | antibiotic inactivation | 159.945 | 158.576 | 151.408 | 156.081 | 154.295 | 154.234 |
| IreK | reduced permeability to antibiotic | 158.214 | 159.745 | 164.275 | 156.308 | 161.382 | 162.909 |
| rpoB2 | antibiotic target alteration; antibiotic target replacement | 172.228 | 173.37 | 172.365 | 179.64 | 175.965 | 174.132 |
| lmrC | antibiotic target protection | 132.888 | 135.901 | 132.801 | 132.392 | 127.341 | 132.299 |
| ugd | antibiotic target alteration | 198.646 | 203.954 | 199.198 | 201.967 | 200.971 | 203.564 |
