# Supplementary material for: Genomic profiling of Elizabethkingia anophelis clinical isolates from a Shanghai hospital: phylogenetic divergence coexists with heterogeneous antibiotic resistance and virulence determinants
Source: Front Microbiol. 2026 Jan 16;16:1751256. doi: 10.3389/fmicb.2025.1751256 (PMC12855470; doi:10.3389/fmicb.2025.1751256)
Supplement: SUPPLEMENTARY Table 2 — Gene Abundance (TPM) of virulence genes across six clinical Elizabethkingia anophelis isolates. [file Table_2.docx]

**Supplementary Table 2. Gene Abundance (TPM) of virulence genes across six clinical Elizabethkingia anophelis isolates**

| **Gene Symbol** | **Virulence Factor Category** | **EA1 (TPM)** | **EA2 (TPM)** | **EA3 (TPM)** | **EA4 (TPM)** | **EA5 (TPM)** | **EA6 (TPM)** |
| --- | --- | --- | --- | --- | --- | --- | --- |
| CT_473 | Effector delivery system | 561.552 | 597.222 | 566.003 | 459.228 | 0 | 545.55 |
| sigE | Regulation | 0 | 0 | 0 | 0 | 308.52 | 0 |
| acpXL | Immune modulation | 705.428 | 827.327 | 664.183 | 992.87 | 771.72 | 902.108 |
| pagR-XO2 | Regulation | 344.105 | 315.619 | 324.133 | 324.85 | 587.349 | 354.534 |
| cesH | Exotoxin | 263.61 | 236.185 | 250.663 | 259.785 | 0 | 240.286 |
| SAUSA300_RS00840 | Immune modulation | 522.991 | 510.717 | 515.546 | 495.764 | 706.478 | 514.271 |
| ROD_RS25695 | Adherence | 415.591 | 410.672 | 392.729 | 406.16 | 576.103 | 360.708 |
| PMI_RS02630 | Adherence | 314.992 | 468.565 | 368.899 | 369.525 | 286.018 | 427.745 |
| ML_RS08565 | Adherence | 427.943 | 424.954 | 539.339 | 498.149 | 572.698 | 415.476 |
| fotS | Adherence | 203.305 | 188.48 | 196.464 | 216.911 | 354.081 | 191.606 |
| algR | Biofilm | 1748.712 | 1809.178 | 1761.681 | 1645.342 | 1678.303 | 1691.344 |
| hopJ1 | Effector delivery system | 423.474 | 539.502 | 496.156 | 491.187 | 392.987 | 414.371 |
| bopD | Biofilm | 959.826 | 883.724 | 950.554 | 998.684 | 929.919 | 1007.044 |
| mprA | Regulation | 1357.332 | 1385.195 | 1413.862 | 1357.965 | 1288.905 | 1364.178 |
| Cj0883c | Motility | 655.602 | 615.443 | 627.557 | 606.875 | 666.461 | 552.45 |
| pagR-XO1 | Regulation | 386.177 | 329.326 | 440.854 | 376.686 | 348.315 | 404.118 |
| adeG | Biofilm | 986.489 | 998.771 | 973.225 | 1025.8 | 943.234 | 917.649 |
| panD | Nutritional/Metabolic factor | 733.544 | 779.46 | 769.419 | 694.756 | 736.466 | 684.856 |
| ahpC | Stress survival | 508.294 | 605.974 | 540.169 | 597.659 | 547.543 | 582.754 |
| btrS | Effector delivery system | 1051.27 | 1112.31 | 1057.31 | 1038.63 | 1019.68 | 1107.85 |
| adeF | Biofilm | 993.421 | 975.481 | 955.567 | 996.611 | 926.09 | 908.258 |
| papX | Adherence | 627.312 | 547.693 | 591.071 | 594.898 | 581.48 | 650.211 |
| sodB | Stress survival | 655.733 | 684.528 | 648.07 | 646.377 | 582.037 | 618.855 |
| fur | Regulation | 680.944 | 702.498 | 667.298 | 761.945 | 670.349 | 692.916 |
| kdtB | Immune modulation | 356.807 | 357.605 | 299.475 | 390.345 | 350.061 | 396.96 |
| pchR | Nutritional/Metabolic factor | 631.88 | 696.894 | 648.038 | 667.279 | 606.604 | 611.993 |
| M3Q_RS01450 | Immune modulation | 305.638 | 364.916 | 372.909 | 305.424 | 363.364 | 379.2 |
| clbP | Exotoxin | 1944.07 | 1978.35 | 1921.79 | 1998.92 | 1933.92 | 1925.67 |
| msrA/B | #N/A | 904.938 | 984.508 | 936.147 | 910.852 | 920.064 | 903.464 |
| ddrA | Immune modulation | 718.452 | 750.94 | 721.296 | 746.13 | 679.9 | 682.85 |
| pglD | Post-translational modification | 179.354 | 253.178 | 205.609 | 174.69 | 229.705 | 199.221 |
| mip | Post-translational modification | 268.2 | 302.598 | 301.404 | 246.272 | 245.628 | 235.179 |
| ricA | Effector delivery system | 520.33 | 547.972 | 576.341 | 579.591 | 518.234 | 573.001 |
| sigL | Regulation | 507.503 | 506.346 | 457.306 | 453.851 | 493.644 | 516.983 |
| ABB77406 | Effector delivery system | 327.468 | 324.162 | 347.887 | 268.389 | 303.749 | 313.023 |
| coxH2/rimL | Effector delivery system | 264.461 | 235.866 | 306.173 | 259.992 | 231.656 | 261.117 |
| ctpV | Nutritional/Metabolic factor | 760.378 | 755.462 | 737.011 | 798.502 | 756.886 | 722.577 |
| tuf | Adherence | 996.959 | 1032.064 | 973.568 | 1043.116 | 1020.636 | 999.798 |
| ideR | Regulation | 198.844 | 240.731 | 205.132 | 198.804 | 162.952 | 187.446 |
| flgJ | Motility | 266.386 | 228.609 | 256.861 | 293.541 | 231.958 | 272.233 |
| iroN | Nutritional/Metabolic factor | 960.657 | 993.418 | 940.708 | 981.654 | 936.55 | 937.217 |
| exsA | Effector delivery system | 1139.655 | 1165.043 | 1194.404 | 1165.912 | 1125.916 | 1172.96 |
| rmlC | Immune modulation | 242.306 | 277.149 | 220.261 | 214.499 | 255.636 | 226.584 |
| flmH | Motility | 2186.228 | 2184.945 | 2174.763 | 2197.64 | 2129.628 | 2170.206 |
| nuc | Exoenzyme | 263.275 | 272.009 | 256.406 | 290.4 | 226.165 | 236.228 |
| fmt | Immune modulation | 414.715 | 394.177 | 425.707 | 391.831 | 374.871 | 363.994 |
| sigM | Regulation | 373.48 | 356.601 | 354.269 | 382.187 | 366.534 | 417.193 |
| pdhB | Adherence | 683.581 | 700.523 | 678.066 | 701.32 | 655.252 | 645.411 |
| iap/cwhA | Invasion | 433.004 | 480.242 | 461.237 | 442.652 | 415.4 | 457.346 |
| wcbR | Immune modulation | 528.046 | 541.456 | 548.356 | 554.103 | 494.46 | 553.756 |
| rfbM | Immune modulation | 332.181 | 322.989 | 317.969 | 341.593 | 277.087 | 305.492 |
| letA | Regulation | 606.761 | 627.962 | 618.807 | 587.813 | 564.974 | 606.97 |
| sugC | Nutritional/Metabolic factor | 415.568 | 401.385 | 453.625 | 436.419 | 412.5 | 395.496 |
| CV_RS12640 | Effector delivery system | 732.697 | 742.319 | 733.181 | 784.506 | 726.26 | 731.197 |
| sadH | Immune modulation | 522.11 | 542.476 | 539.94 | 580.854 | 527.828 | 557.501 |
| omp89 | Adherence | 175.74 | 186.96 | 164.585 | 224.561 | 171.132 | 179.327 |
| cesC | Exotoxin | 567.804 | 563.703 | 589.666 | 607.604 | 548.887 | 565.091 |
| algU | Biofilm | 256.553 | 281.636 | 295.215 | 287.287 | 274.097 | 318.152 |
| sbnA | Nutritional/Metabolic factor | 493.471 | 529.125 | 473.581 | 490.13 | 518.681 | 489.714 |
| ompA | Immune modulation | 544.813 | 559.064 | 532.299 | 537.18 | 500.853 | 517.192 |
| YPO_RS03555 | Effector delivery system | 241.297 | 259.204 | 241.298 | 250.311 | 199.658 | 238.33 |
| mtrC | Antimicrobial activity/Competitive advantage | 605.551 | 631.866 | 636.279 | 602.952 | 588.066 | 592.299 |
| aslA | Invasion | 686.748 | 679.304 | 634.004 | 671.531 | 649.863 | 655.952 |
| MGA_RS01700 | Adherence | 277.648 | 255.138 | 286.388 | 308.934 | 291.674 | 263.933 |
| tolC | Nutritional/Metabolic factor | 492.926 | 531.676 | 495.528 | 535.413 | 499.724 | 499.526 |
| C8J_RS05595 | Immune modulation | 311.186 | 308.499 | 313.587 | 268.823 | 278.622 | 304.046 |
| legP | Effector delivery system | 312.557 | 309.726 | 313.013 | 334.133 | 310.561 | 276.723 |
| hrpY | Effector delivery system | 192.164 | 222.541 | 173.946 | 191.014 | 189.905 | 215.912 |
| clbD | Exotoxin | 659.362 | 676.412 | 690.031 | 669.705 | 653.049 | 639.168 |
| farA | Antimicrobial activity/Competitive advantage | 524.245 | 538.993 | 498.255 | 541.999 | 539.663 | 512.124 |
| cadF | Adherence | 382.459 | 387.511 | 375.358 | 416.667 | 378.268 | 411.755 |
| fepA | Nutritional/Metabolic factor | 685.683 | 697.113 | 657.046 | 706.211 | 693.996 | 700.324 |
| qbsC | Nutritional/Metabolic factor | 545.6 | 511.356 | 495.889 | 532.64 | 515.055 | 525.774 |
| farB | Antimicrobial activity/Competitive advantage | 816.344 | 814.866 | 778.361 | 806.423 | 816.979 | 783.684 |
| rmlB | Immune modulation | 186.626 | 207.062 | 202.213 | 179.514 | 159.969 | 178.778 |
| phzF1 | Nutritional/Metabolic factor | 204.3 | 188.23 | 195.567 | 177.661 | 208.414 | 162.635 |
| kpsU | Invasion | 172.171 | 171.62 | 198.248 | 185.513 | 192.642 | 151.86 |
| chuD | Nutritional/Metabolic factor | 207.262 | 207.77 | 173.888 | 196.285 | 170.006 | 178.684 |
| leuD | Nutritional/Metabolic factor | 217.915 | 211.455 | 241.704 | 214.954 | 211.973 | 189.253 |
| mpa | Stress survival | 543.586 | 532.729 | 497.99 | 519.058 | 520.458 | 505.971 |
| STER_RS07085 | Immune modulation | 247.807 | 264.213 | 227.248 | 273.363 | 251.029 | 241.313 |
| hopI1 | Effector delivery system | 244.093 | 217.259 | 215.084 | 217.291 | 199.906 | 199.119 |
| phnB | Effector delivery system | 266.092 | 276.532 | 279.556 | 253.362 | 240.735 | 281.544 |
| KVAR_RS26115 | Immune modulation | 211.763 | 233.07 | 249.883 | 204.111 | 222.754 | 227.767 |
| clpP | Stress survival | 207.216 | 228.395 | 214.684 | 191.163 | 195.898 | 229.871 |
| argK | Exotoxin | 207.577 | 205.92 | 213.14 | 233.159 | 219.955 | 246.727 |
| ndk | Immune modulation | 280.056 | 273.239 | 295.466 | 300.477 | 258.019 | 270.697 |
| fimZ | Adherence | 198.31 | 194.676 | 199.233 | 181.472 | 157.503 | 191.037 |
| PA4709 | Nutritional/Metabolic factor | 166.265 | 199.13 | 167.73 | 182.186 | 159.183 | 158.485 |
| cbrD | Nutritional/Metabolic factor | 161.674 | 141.417 | 137.31 | 165.402 | 139.142 | 124.911 |
| EAMY_RS32255 | Nutritional/Metabolic factor | 355.121 | 338.71 | 369.582 | 362.158 | 334.769 | 332.839 |
| pvdM | Nutritional/Metabolic factor | 187.111 | 206.349 | 178.428 | 191.913 | 159.929 | 179.649 |
| crc | Adherence | 202.286 | 209.086 | 229.919 | 237.462 | 200.152 | 209.754 |
| pvdO | Nutritional/Metabolic factor | 171.367 | 195.408 | 193.036 | 197.116 | 186.509 | 159.476 |
| dnaK | Adherence | 411.287 | 414.526 | 382.111 | 416.882 | 418.356 | 425.444 |
| vfr | Adherence | 450.863 | 479.668 | 448.04 | 467.297 | 481.544 | 479.019 |
| rfaE1 | Immune modulation | 469.768 | 448.204 | 447.87 | 424.626 | 447.96 | 459.321 |
| ABZJ_RS06220 | Immune modulation | 212.631 | 212.73 | 182.053 | 180.304 | 185.255 | 200.031 |
| Cj1437c | Immune modulation | 491.69 | 516.431 | 498.024 | 518.607 | 484.087 | 486.453 |
| algZ | Biofilm | 1136.614 | 1140.164 | 1099.199 | 1115.238 | 1123.246 | 1125.957 |
| hisF2 | Immune modulation | 210.926 | 190.643 | 167.772 | 198.416 | 202.009 | 187.703 |
| hisH2 | Immune modulation | 226.936 | 220.535 | 225.512 | 198.673 | 192.83 | 203.76 |
| DDA3937_RS14700 | Nutritional/Metabolic factor | 443.538 | 441.935 | 422.34 | 463.615 | 444.243 | 426.554 |
| irtB | Nutritional/Metabolic factor | 274.801 | 271.652 | 260.399 | 303.682 | 275.387 | 269.159 |
| lisK | Regulation | 310.601 | 281.852 | 309.685 | 294.701 | 276.678 | 284.275 |
| PMI_RS06910 | Nutritional/Metabolic factor | 156.989 | 198.978 | 178.475 | 183.991 | 169.683 | 168.726 |
| mtrE | Antimicrobial activity/Competitive advantage | 279.075 | 284.074 | 274.178 | 282.001 | 266.903 | 245.454 |
| phoR | Regulation | 333.3 | 332.54 | 325.771 | 307.031 | 302.93 | 304.892 |
| chpD | Adherence | 841.736 | 844.085 | 813.15 | 830.336 | 817.665 | 812.041 |
| cylG | Exotoxin | 213.074 | 214.889 | 185.416 | 227.508 | 201.095 | 206.742 |
| hemA | Nutritional/Metabolic factor | 224.29 | 208.759 | 194.056 | 234.173 | 211.663 | 223.058 |
| allB | Nutritional/Metabolic factor | 188.253 | 195.716 | 181.858 | 192.518 | 166.06 | 207.287 |
| CJJ81176_RS05515 | Immune modulation | 204.371 | 220.783 | 190.774 | 185.023 | 209.029 | 189.724 |
| pilR | Adherence | 479.395 | 484.243 | 456.421 | 491.8 | 461.026 | 470.731 |
| wbfT | Immune modulation | 374.863 | 356.771 | 380.848 | 373.559 | 344.759 | 373.527 |
| wbpE | Immune modulation | 171.116 | 158.603 | 164.09 | 157.8 | 156.926 | 130.895 |
| flhF | Motility | 374.39 | 381.004 | 364.314 | 368.699 | 346.487 | 349.849 |
| mtrD | Antimicrobial activity/Competitive advantage | 307.799 | 320.556 | 289.529 | 320.849 | 296.02 | 295.09 |
| LPG_RS14840 | Effector delivery system | 228.464 | 222.464 | 239.694 | 240.135 | 203.474 | 223.521 |
| phzE1 | Nutritional/Metabolic factor | 187.233 | 198.235 | 190.325 | 193.971 | 185.663 | 222.267 |
| EFMU0317_RS16950 | Adherence | 313.807 | 292.414 | 309.027 | 279.738 | 286.02 | 288.64 |
| CBUD_RS12405 | Effector delivery system | 391.277 | 410.631 | 412.564 | 425.8 | 399.48 | 392.009 |
| mig-5 | Antimicrobial activity/Competitive advantage | 263.927 | 287.017 | 269.708 | 292.669 | 274.725 | 297.618 |
| STR_RS05220 | Immune modulation | 140.807 | 168.273 | 175.571 | 146.729 | 159.137 | 166.987 |
| hitC | Nutritional/Metabolic factor | 436.993 | 429.472 | 456.242 | 454.415 | 441.925 | 422.426 |
| wbmP | Immune modulation | 144.306 | 174.717 | 166.419 | 142.012 | 167.392 | 154.794 |
| icaR | Biofilm | 225.923 | 203.375 | 242.099 | 215.628 | 220.983 | 231.292 |
| waaZ | Immune modulation | 150.498 | 163.572 | 163.572 | 142.728 | 132.789 | 163.939 |
| SAK_RS06335 | Immune modulation | 196.429 | 205.02 | 217.448 | 218.16 | 213.847 | 235.026 |
| mrfI | Adherence | 411.326 | 388.527 | 373.623 | 401.653 | 397.887 | 388.1 |
| bioA | Nutritional/Metabolic factor | 176.667 | 181.24 | 170.548 | 177.189 | 156.417 | 148.849 |
| BT9727_RS25815 | Immune modulation | 179.407 | 203.262 | 184.438 | 171.479 | 180.442 | 202.091 |
| gmd | Immune modulation | 180.193 | 203.614 | 194.203 | 212.85 | 180.928 | 199.927 |
| acrB | Antimicrobial activity/Competitive advantage | 435.694 | 436.021 | 420.402 | 440.075 | 415.631 | 408.598 |
| basJ | Nutritional/Metabolic factor | 194.019 | 214.691 | 195.48 | 194.629 | 225.096 | 200.271 |
| exbB | Nutritional/Metabolic factor | 240.305 | 239.141 | 226.32 | 262.286 | 249.029 | 231.34 |
| phzG1 | Nutritional/Metabolic factor | 259.96 | 286.193 | 255.246 | 251.497 | 271.228 | 259.357 |
| SMUNN2025_RS06365 | Biofilm | 179.317 | 183.508 | 191.05 | 176.924 | 193.68 | 211.766 |
| sodC | Stress survival | 324.561 | 361.44 | 350.307 | 336.563 | 349.99 | 343.834 |
| entA | Nutritional/Metabolic factor | 229.816 | 208.018 | 206.026 | 209.645 | 232.606 | 204.191 |
| ASA_RS06915 | Motility | 195.768 | 209.57 | 212.65 | 201.295 | 182.668 | 183.831 |
| sigH | Regulation | 827.613 | 799.079 | 793.793 | 804.575 | 811.286 | 818.796 |
| hdtS | Biofilm | 185.151 | 201.621 | 169.803 | 199.509 | 199.511 | 183.742 |
| BJAB0715_RS05230 | Nutritional/Metabolic factor | 292.988 | 286.524 | 277.195 | 280.479 | 267.562 | 303.19 |
| rpe | Immune modulation | 160.398 | 184.65 | 180.886 | 161.76 | 153.324 | 162.65 |
| chuV | Nutritional/Metabolic factor | 183.863 | 176.944 | 195.975 | 191.328 | 161.147 | 175.922 |
| bplG | Immune modulation | 209.272 | 231.966 | 232.276 | 215.695 | 201.126 | 214.953 |
| kasB | Nutritional/Metabolic factor | 507.543 | 499.8 | 506.483 | 506.325 | 474.956 | 495.339 |
| oatA | Immune modulation | 487.097 | 478.912 | 485.117 | 499.975 | 503.728 | 471.198 |
| mgtC | Nutritional/Metabolic factor | 383.475 | 377.9 | 395.746 | 396.551 | 369.415 | 368.508 |
| mbtN | Nutritional/Metabolic factor | 327.025 | 326.932 | 300.547 | 332.999 | 316.817 | 311.665 |
| ibeB | Invasion | 672.378 | 667.166 | 653.4 | 690.039 | 676.471 | 673.504 |
| ETAE_RS04230 | Effector delivery system | 166.485 | 184.85 | 164.857 | 151.705 | 166.538 | 153.141 |
| gluP | Immune modulation | 539.957 | 534.666 | 509.622 | 527.709 | 536.259 | 517.742 |
| lpxB | Immune modulation | 181.624 | 180.898 | 162.602 | 198.724 | 182.07 | 175.612 |
| rmlD | Immune modulation | 209.98 | 238.861 | 206.474 | 219.189 | 222.799 | 225.59 |
| manC | Immune modulation | 174.379 | 177.456 | 173.174 | 168.544 | 146.681 | 159.483 |
| carA | Nutritional/Metabolic factor | 300.979 | 318.404 | 299.204 | 319.721 | 292.646 | 297.077 |
| orfM | Immune modulation | 182.43 | 201.766 | 192.717 | 188.931 | 178.155 | 169.168 |
| ABZJ_RS06255 | Immune modulation | 148.64 | 147.389 | 155.027 | 155.399 | 134.108 | 127.437 |
| adhD | Immune modulation | 328.654 | 329.537 | 335.206 | 349.769 | 315.035 | 327.046 |
| SAG_RS08560 | Immune modulation | 210.5 | 206.82 | 196.848 | 184.128 | 184.256 | 203.569 |
| pvdN | Nutritional/Metabolic factor | 203.95 | 201.726 | 200.177 | 230.589 | 212.319 | 208.303 |
| wcbP | Immune modulation | 234.37 | 258.192 | 232.836 | 257.472 | 241.9 | 238.968 |
| atsB | Effector delivery system | 145.981 | 160.692 | 150.903 | 147.935 | 139.304 | 170.445 |
| BQ_RS02100 | Nutritional/Metabolic factor | 167.145 | 173.148 | 166.585 | 168.767 | 141.804 | 159.668 |
| brkB | Immune modulation | 189.254 | 195.13 | 166.124 | 181.048 | 186.764 | 170.947 |
| LPG_RS11860 | Effector delivery system | 375.601 | 364.376 | 373.207 | 371.659 | 363.879 | 345.177 |
| lpxC | Immune modulation | 167.441 | 157.586 | 145.091 | 160.909 | 151.593 | 137.031 |
| sctN | Effector delivery system | 349.193 | 363.62 | 341.315 | 356.17 | 355.185 | 333.246 |
| purC | Nutritional/Metabolic factor | 167.523 | 159.816 | 167.129 | 165.254 | 141.489 | 148.083 |
| devR/dosR | Regulation | 169.483 | 177.319 | 185.991 | 177.892 | 194.322 | 164.506 |
| SAG_RS08545 | Immune modulation | 172.953 | 180.414 | 163.508 | 164.99 | 191.816 | 180.332 |
| panC | Nutritional/Metabolic factor | 195.991 | 176.657 | 193.613 | 180.626 | 168.318 | 176.905 |
| katA | Stress survival | 189.798 | 206.177 | 194.338 | 218.619 | 208.745 | 196.679 |
| fadD13 | Immune modulation | 445.291 | 464.237 | 464.952 | 460.632 | 441.403 | 464.978 |
| syrB1 | Exotoxin | 146.274 | 154.066 | 133.468 | 124.922 | 139.084 | 148.164 |
| YE105_RS01775 | Effector delivery system | 381.754 | 367.457 | 359.81 | 368.905 | 361.449 | 385.785 |
| plr/gapA | Adherence | 198.608 | 213.673 | 187.681 | 202.936 | 201.229 | 217.064 |
| bioD | Nutritional/Metabolic factor | 230.37 | 245.665 | 237.821 | 234.261 | 220.893 | 217.167 |
| bplF | Immune modulation | 144.432 | 168.949 | 152.273 | 140.906 | 150.748 | 162.134 |
| dhbA | Nutritional/Metabolic factor | 217.375 | 232.234 | 210.934 | 237.349 | 217.177 | 231.139 |
| relA | Regulation | 351.327 | 352.935 | 344.891 | 366.154 | 347.155 | 370.817 |
| LPG_RS03775 | Immune modulation | 187.419 | 191.895 | 168.178 | 186.428 | 166.885 | 181.748 |
| iraB | Nutritional/Metabolic factor | 451.427 | 455.295 | 434.972 | 437.716 | 427.582 | 437.675 |
| SAK_RS06285 | Immune modulation | 157.354 | 177.689 | 150.67 | 149.536 | 164.878 | 162.938 |
| bplA | Nutritional/Metabolic factor | 406.392 | 429.908 | 402.888 | 421.454 | 406.885 | 413.262 |
| fleQ | Motility | 188.737 | 193.702 | 179.466 | 205.282 | 200.142 | 206.276 |
| IlpA | Adherence | 167.389 | 159.772 | 183.912 | 179.794 | 164.579 | 159.435 |
| purM | Nutritional/Metabolic factor | 180.379 | 172.264 | 176.063 | 188.828 | 169.045 | 158.454 |
| napA | Immune modulation | 284.465 | 275.571 | 287.824 | 291.547 | 269.787 | 297.729 |
| hspX | Stress survival | 319.327 | 335.492 | 319.101 | 306.166 | 325.824 | 313.054 |
| aut | Invasion | 181.682 | 185.052 | 191.622 | 197.434 | 167.838 | 181.39 |
| CBU_1566 | Effector delivery system | 220.923 | 240.925 | 239.924 | 236.209 | 252.238 | 237.765 |
| vabA | Nutritional/Metabolic factor | 179.439 | 166.994 | 180.545 | 161.71 | 179.905 | 158.209 |
| aatC | Others | 185.31 | 157.655 | 181.538 | 181.076 | 181.788 | 179.137 |
| fpvA | Nutritional/Metabolic factor | 311.15 | 298.981 | 296.725 | 316.639 | 301.897 | 288.962 |
| nuoG | Immune modulation | 217.012 | 203.22 | 193.642 | 217.161 | 211.341 | 197.654 |
| PSPTO_RS07240 | Effector delivery system | 177.69 | 164.242 | 156.217 | 163.588 | 148.688 | 155.571 |
| pchA | Nutritional/Metabolic factor | 170.517 | 175.474 | 176.634 | 153.577 | 169.609 | 155.474 |
| KVAR_RS26125 | Immune modulation | 189.641 | 186.363 | 168.067 | 191.828 | 172.26 | 175.23 |
| DDA3937_RS07730 | Nutritional/Metabolic factor | 157.25 | 157.892 | 152.186 | 154.387 | 131.347 | 146.968 |
| ligA | Adherence | 159.777 | 150.33 | 134.894 | 163.616 | 152.945 | 150.048 |
| pdgA | Immune modulation | 178.927 | 179.096 | 168.848 | 173.398 | 184.497 | 156.723 |
| YPK_RS15930 | Immune modulation | 172.672 | 183.302 | 196.169 | 174.095 | 168.711 | 177.836 |
| vpdC | Effector delivery system | 333.667 | 339.635 | 311.571 | 326.572 | 334.361 | 326.298 |
| lpxA | Immune modulation | 194.448 | 188.303 | 199.58 | 202.783 | 198.058 | 175.94 |
| pgi | Immune modulation | 202.489 | 206.461 | 189.888 | 219.826 | 206.719 | 200.646 |
| M3Q_RS01475 | Immune modulation | 187.62 | 191.51 | 184.634 | 188.59 | 168.548 | 197.275 |
| EF0818 | Exoenzyme | 313.755 | 313.066 | 296.363 | 324.448 | 305.269 | 304.365 |
| fimE | Adherence | 215.376 | 222.153 | 205.064 | 234.386 | 219.73 | 215.508 |
| wbkC | Immune modulation | 197.817 | 201.718 | 224.728 | 210.651 | 214.691 | 213.319 |
| clbF | Exotoxin | 306.487 | 298.151 | 303.754 | 313.295 | 286.315 | 293.71 |
| cpsB/cdsA | Immune modulation | 215.743 | 223.995 | 212.546 | 205.604 | 203.078 | 197.359 |
| kdsA | Immune modulation | 333.869 | 330.477 | 348.224 | 334.055 | 321.461 | 323.478 |
| MCR_RS07635 | Adherence | 203.161 | 190.907 | 208.013 | 207.142 | 186.596 | 189.865 |
| cpsA/uppS | Immune modulation | 245.429 | 270.033 | 261.54 | 261.598 | 248.142 | 263.579 |
| acpC | Stress survival | 167.346 | 172.505 | 189.919 | 189.197 | 171.61 | 179.617 |
| zmp1 | Exoenzyme | 319.228 | 307.993 | 303.332 | 294.257 | 300.12 | 294.108 |
| lpxH | Immune modulation | 206.521 | 197.436 | 205.997 | 184.418 | 186.886 | 191.102 |
| PSYR_RS13390 | Nutritional/Metabolic factor | 205.975 | 190.67 | 185.804 | 204.672 | 210.242 | 200.843 |
| bcr | Nutritional/Metabolic factor | 285.231 | 284.401 | 278.657 | 292.637 | 279.3 | 264.373 |
| clpC | Stress survival | 325.952 | 325.689 | 318.781 | 342.715 | 321.582 | 315.988 |
| bioB | Nutritional/Metabolic factor | 171.529 | 178.799 | 177.19 | 191.833 | 163.349 | 179.035 |
| fxbA | Nutritional/Metabolic factor | 192.517 | 176.262 | 187.326 | 194.335 | 171.706 | 177.422 |
| C_RS11765 | Nutritional/Metabolic factor | 473.01 | 465.727 | 454.267 | 467.914 | 464.074 | 447.682 |
| qbsM | Nutritional/Metabolic factor | 160.826 | 163.757 | 156.995 | 179.229 | 154.571 | 154.382 |
| lirB | Effector delivery system | 151.736 | 165.747 | 151.311 | 147.556 | 146.44 | 137.068 |
| vpsH | Biofilm | 174.255 | 188.083 | 175.569 | 198.52 | 187.104 | 178.464 |
| tke10 | Effector delivery system | 137.959 | 137.977 | 133.291 | 145.454 | 117.756 | 137.021 |
| CBU_2076 | Effector delivery system | 147.461 | 153.253 | 156.867 | 157.028 | 132.627 | 144.958 |
| phzS | Nutritional/Metabolic factor | 151.924 | 174.213 | 164.997 | 171.633 | 152.902 | 163.728 |
| pdtorfO | Nutritional/Metabolic factor | 285.557 | 297.99 | 295.14 | 300.554 | 283.753 | 277.013 |
| pat2 | Exotoxin | 147.968 | 146.999 | 165.569 | 140.506 | 156.767 | 143.855 |
| CFF8240_RS06905 | Immune modulation | 211.881 | 195.665 | 218.089 | 208.433 | 195.815 | 211.996 |
| CJE_RS07975 | Immune modulation | 163.112 | 176.604 | 176.32 | 151.845 | 166.826 | 167.602 |
| wecA | Immune modulation | 151.048 | 166.925 | 163.959 | 167.481 | 152.218 | 174.158 |
| gtrB | Immune modulation | 310.409 | 308.498 | 313.586 | 318.138 | 314.94 | 292.312 |
| clbG | Exotoxin | 220.499 | 200.205 | 209.438 | 219.994 | 210.273 | 199.913 |
| SH_RS01845 | Immune modulation | 160.138 | 164.957 | 155.628 | 146.388 | 143.34 | 163.103 |
| BMA_RS19520 | Effector delivery system | 236.246 | 236.751 | 247.811 | 240.768 | 255.288 | 256.053 |
| mucD | Biofilm | 181.3 | 194.408 | 182.816 | 200.721 | 179.081 | 180.454 |
| acrA | Antimicrobial activity/Competitive advantage | 378.921 | 371.998 | 374.46 | 394.643 | 379.954 | 370.267 |
| kdtA/waaA | Immune modulation | 173.932 | 180.485 | 179.846 | 199.771 | 184.425 | 180.767 |
| trpD | Nutritional/Metabolic factor | 165.423 | 158.531 | 139.506 | 154.742 | 155.93 | 149.576 |
| SSU05_0574 | Immune modulation | 173.612 | 169.336 | 182.626 | 161.778 | 172.856 | 158.456 |
| pilS | Adherence | 162.376 | 173.368 | 150.24 | 166.285 | 155.548 | 153.45 |
| hmuU | Nutritional/Metabolic factor | 150.376 | 137.788 | 152.325 | 136.668 | 140.315 | 129.491 |
| lpxD2 | Immune modulation | 165.154 | 161.675 | 157.31 | 145.063 | 145.912 | 148.052 |
| SMU_RS01255 | Immune modulation | 207.94 | 201.554 | 200.166 | 217.939 | 195.653 | 213.973 |
| msbA | Immune modulation | 358.142 | 366.541 | 342.386 | 359.678 | 348.562 | 352.713 |
| prsA2 | Post-translational modification | 154.602 | 157.836 | 169.448 | 154.45 | 162.413 | 143.874 |
| pvuA | Nutritional/Metabolic factor | 138.002 | 145.612 | 133.699 | 128.78 | 146.605 | 126.02 |
| tviB | Immune modulation | 165.779 | 156.989 | 162.951 | 153.063 | 143.623 | 164.866 |
| tufA | Adherence | 220.321 | 222.732 | 215.304 | 236.767 | 232.509 | 217.978 |
| pdtorfM | Nutritional/Metabolic factor | 190.171 | 213.583 | 196.332 | 208.594 | 204.159 | 199.978 |
| senX3 | Regulation | 479.697 | 495.396 | 472.552 | 492.839 | 484.557 | 487.365 |
| ylxH | Motility | 171.749 | 181.127 | 187.997 | 165.65 | 169.067 | 179.291 |
| rfbD | Immune modulation | 228.653 | 223.272 | 210.18 | 232.839 | 217.58 | 216.757 |
| lpxD | Immune modulation | 182.05 | 198.855 | 192.655 | 185.08 | 177.208 | 179.249 |
| STER_RS07090 | Immune modulation | 145.974 | 167.471 | 156.747 | 168.556 | 156.962 | 156.754 |
| coxH3 | Effector delivery system | 179.977 | 177.377 | 179.054 | 191.613 | 165.484 | 179.166 |
| nagJ | Exoenzyme | 314.269 | 307.307 | 297.228 | 315.896 | 303.816 | 296.263 |
| lsgC | Immune modulation | 171.236 | 174.188 | 171.878 | 157.46 | 173.198 | 183.218 |
| BC_RS26290 | Immune modulation | 157.657 | 166.653 | 153.344 | 152.268 | 163.642 | 173.573 |
| hemB | Nutritional/Metabolic factor | 200.843 | 185.11 | 191.206 | 200.438 | 180.257 | 188.465 |
| KPN2242_RS16285 | Immune modulation | 155.751 | 175.727 | 152.967 | 156.034 | 161.1 | 163.98 |
| hmuV | Nutritional/Metabolic factor | 334.027 | 331.137 | 320.101 | 327.763 | 328.018 | 311.465 |
| tle1 | Effector delivery system | 148.088 | 154.739 | 146.669 | 149.351 | 143.956 | 130.428 |
| bioF | Nutritional/Metabolic factor | 176.258 | 183.637 | 163.849 | 165.748 | 181.828 | 171.6 |
| hemE | Nutritional/Metabolic factor | 202.952 | 209.931 | 208.967 | 224.247 | 221.843 | 215.791 |
| mucP | Biofilm | 200.703 | 200.042 | 185.889 | 210.822 | 199.828 | 204.042 |
| gndA | Immune modulation | 146.362 | 164.651 | 145.941 | 143.638 | 144.972 | 143.949 |
| JJD26997_RS08170 | Immune modulation | 138.501 | 152.379 | 142.093 | 127.723 | 135.973 | 141.189 |
| manA | Immune modulation | 167.735 | 177.518 | 167.394 | 184.952 | 162.914 | 169.22 |
| glgA | Effector delivery system | 143.491 | 146.421 | 125.217 | 139.85 | 137.694 | 146.905 |
| tssI | Effector delivery system | 280.246 | 277.247 | 269.677 | 280.781 | 271.15 | 259.668 |
| lysA | Nutritional/Metabolic factor | 215.586 | 203.656 | 191.3 | 197.872 | 202.848 | 202.957 |
| tig/ropA | Stress survival | 197.499 | 190.648 | 184.543 | 199.101 | 182.424 | 180.115 |
| yycJ | Effector delivery system | 160.882 | 162.343 | 177.001 | 153.876 | 169.861 | 165.82 |
| hrpX | Effector delivery system | 141.374 | 138.483 | 138.769 | 141.014 | 120.655 | 133.365 |
| LLO_RS11895 | Others | 156.805 | 168.885 | 174.628 | 165.407 | 158.922 | 154.291 |
| pat1 | Exotoxin | 185.355 | 188.082 | 171.94 | 194.473 | 188.191 | 191.145 |
| PMI_RS01140 | Nutritional/Metabolic factor | 145.398 | 163.081 | 154.313 | 157.632 | 158.19 | 143.249 |
| iutA | Nutritional/Metabolic factor | 179.446 | 175.53 | 170.409 | 176.161 | 164.391 | 186.978 |
| hcnC | Antimicrobial activity/Competitive advantage | 147.298 | 131.843 | 132.334 | 143.031 | 127.571 | 131.66 |
| mps1 | Immune modulation | 289.877 | 299.849 | 289.807 | 300.896 | 291.161 | 279.916 |
| purCD | Nutritional/Metabolic factor | 152.921 | 153.314 | 150.902 | 155.423 | 150.361 | 134.252 |
| endoS | Immune modulation | 176.972 | 170.201 | 170.776 | 183.489 | 182.363 | 163.812 |
| cyaB | Exotoxin | 471.563 | 481.993 | 461.122 | 462.23 | 468.874 | 472.521 |
| hemH | Nutritional/Metabolic factor | 204.773 | 199.911 | 199.993 | 196.107 | 212.139 | 215.473 |
| qbsN | Nutritional/Metabolic factor | 178.472 | 177.625 | 171.106 | 188.639 | 178.474 | 191.461 |
| vabS | Nutritional/Metabolic factor | 181.686 | 171.435 | 178.899 | 169.977 | 166.61 | 186.095 |
| feoB | Nutritional/Metabolic factor | 198.63 | 190.415 | 177.933 | 194.146 | 197.211 | 188.214 |
| STER_RS05260 | Immune modulation | 161.089 | 174.052 | 171.392 | 154.648 | 158.425 | 163.074 |
| carB | Nutritional/Metabolic factor | 143.892 | 149.645 | 136.558 | 150.364 | 140.145 | 131.182 |
| wbtH | Immune modulation | 163.729 | 172.478 | 161.85 | 157.358 | 150.106 | 157.847 |
| bspR2 | Biofilm | 158.595 | 142.451 | 164.561 | 152.985 | 150.8 | 152.266 |
| KPR_RS08985 | Immune modulation | 148.365 | 138.735 | 130.965 | 140.209 | 127.461 | 134.336 |
| galE | Immune modulation | 311.576 | 293.59 | 311.779 | 298.025 | 307.018 | 301.885 |
| irgA | Nutritional/Metabolic factor | 153.232 | 140.894 | 143.503 | 146.108 | 143.551 | 130.555 |
| gmhB | Immune modulation | 155.901 | 148.475 | 142.972 | 144.335 | 134.224 | 140.282 |
| ASA_RS16545 | Nutritional/Metabolic factor | 184.457 | 176.202 | 179.058 | 168.58 | 167.813 | 184.277 |
| btpA | Immune modulation | 128.249 | 139.675 | 142.883 | 147.476 | 144.855 | 133.739 |
| nagL | Exoenzyme | 151.385 | 143.202 | 147.626 | 151.408 | 148.393 | 132.299 |
| pdxA | Motility | 206.858 | 196.556 | 189.641 | 201.431 | 187.758 | 199.106 |
| iroE | Nutritional/Metabolic factor | 145.112 | 158.105 | 148.718 | 163.864 | 160.895 | 154.472 |
| adeH | Biofilm | 137.067 | 146.133 | 135.11 | 142.766 | 135.699 | 125.251 |
| rfaF | Immune modulation | 198.842 | 205.469 | 205.226 | 199.928 | 211.57 | 217.868 |
| hlyA | Exotoxin | 185.268 | 184.603 | 177.583 | 185.013 | 168.633 | 172.723 |
| fpvR | Nutritional/Metabolic factor | 210.961 | 215.623 | 209.635 | 225.051 | 226.953 | 216.717 |
| ASA_RS16540 | Nutritional/Metabolic factor | 133.428 | 130.714 | 125.122 | 129.88 | 125.453 | 113.284 |
| QU43_RS38280 | Effector delivery system | 184.904 | 169.79 | 173.771 | 165.417 | 166.036 | 171.823 |
| pdxJ | Motility | 195.569 | 208.923 | 211.254 | 193.909 | 206.102 | 202.461 |
| ABK1_RS00450 | Immune modulation | 171.024 | 176.099 | 180.85 | 184.577 | 172.871 | 164.844 |
| C8J_RS05615 | Immune modulation | 184.693 | 194.723 | 192.564 | 186.519 | 189.214 | 174.916 |
| viuB | Nutritional/Metabolic factor | 163.065 | 163.28 | 161.063 | 177.637 | 160.767 | 157.854 |
| LPG_RS03855 | Immune modulation | 145.065 | 144.269 | 159.403 | 143.184 | 140.878 | 140.857 |
| sigA/rpoV | Regulation | 154.272 | 147.787 | 153.301 | 140.912 | 142.787 | 158.603 |
| algC | Biofilm | 212.032 | 215.092 | 199.297 | 214.115 | 201.687 | 213.645 |
| ptxR | Nutritional/Metabolic factor | 146.765 | 163.77 | 152.096 | 155.404 | 152.262 | 164.098 |
| fagC | Nutritional/Metabolic factor | 356.324 | 366.624 | 354.101 | 372.108 | 366.382 | 359.861 |
| hemN | Nutritional/Metabolic factor | 344.049 | 338.296 | 336.772 | 336.243 | 323.435 | 332.984 |
| PSPTO_RS22515 | Exotoxin | 155.827 | 145.108 | 142.368 | 157.738 | 142.341 | 151.926 |
| hxuC | Nutritional/Metabolic factor | 135.492 | 131.956 | 128.495 | 138.671 | 134.247 | 119.299 |
| YPO_RS02285 | Effector delivery system | 157.075 | 167.935 | 152.209 | 164.79 | 152.403 | 153.386 |
| chuA | Nutritional/Metabolic factor | 183.213 | 188.748 | 173.999 | 192.76 | 190.498 | 184.556 |
| EAMY_RS32250 | Nutritional/Metabolic factor | 133.976 | 143.895 | 124.554 | 128.14 | 134.632 | 136.37 |
| clpE | Stress survival | 172.734 | 170.511 | 173.013 | 176.294 | 169.047 | 187.642 |
| lgt | Post-translational modification | 308.787 | 290.316 | 302.333 | 293.939 | 301.533 | 302.406 |
| ppk1 | Adherence | 168.029 | 156.9 | 157.75 | 173.303 | 161.544 | 158.17 |
| hlyB | Exotoxin | 308.107 | 306.825 | 296.843 | 299.784 | 290.681 | 296.826 |
| pyrB | Nutritional/Metabolic factor | 168.635 | 177.311 | 169.072 | 164.544 | 177.432 | 161.151 |
| CBU_0270 | Effector delivery system | 163.717 | 144.444 | 152.641 | 152.434 | 155.916 | 159.061 |
| wbuZ | Immune modulation | 175.984 | 189.752 | 193.105 | 183.424 | 181.003 | 179.05 |
| YPTB_RS05510 | Immune modulation | 207.465 | 210.888 | 197.319 | 203.553 | 194.963 | 196.463 |
| hemL | Nutritional/Metabolic factor | 187.817 | 187.913 | 183.991 | 175.202 | 172.576 | 179.091 |
| eno | Exoenzyme | 193.986 | 193.251 | 192.491 | 206.918 | 189.4 | 189.748 |
| nanJ | Exoenzyme | 264.25 | 284.027 | 274.739 | 275.567 | 272.874 | 271.373 |
| fslC | Nutritional/Metabolic factor | 162.287 | 169.316 | 157.704 | 170.442 | 174.059 | 172.858 |
| eptC | Motility | 127.144 | 137.351 | 139.672 | 145.582 | 137.85 | 131.672 |
| hpuB | Nutritional/Metabolic factor | 180.482 | 183.709 | 173.164 | 191.031 | 188.975 | 181.954 |
| rmlA | Immune modulation | 172.075 | 180.294 | 184.556 | 184.804 | 187.208 | 173.226 |
| icl | Others | 187.971 | 184.898 | 172.114 | 177.754 | 188.195 | 180.832 |
| coxU2 | Effector delivery system | 134.246 | 138.486 | 129.62 | 139.795 | 122.906 | 135.835 |
| PEB4 | Biofilm | 180.152 | 177.874 | 178.326 | 188.088 | 186.157 | 193.412 |
| prrB | Regulation | 138.043 | 120.273 | 124.793 | 125.376 | 125.947 | 122.213 |
| manB/yhxB | Immune modulation | 137.6 | 138.968 | 133.851 | 130.72 | 146.585 | 129.869 |
| PSPA7_RS09480 | Immune modulation | 135.194 | 151.201 | 137.256 | 137.472 | 138.567 | 145.409 |
| gadC | Nutritional/Metabolic factor | 131.616 | 126.942 | 134.693 | 139.968 | 122.813 | 127.738 |
| rfbB | Immune modulation | 157.587 | 173.873 | 170.934 | 171.44 | 172.17 | 173.309 |
| BJAB0868_RS00505 | Immune modulation | 228.956 | 221.439 | 220.906 | 236.687 | 222.301 | 224.834 |
| DDA3937_RS07710 | Nutritional/Metabolic factor | 178.517 | 174.551 | 172.403 | 163.713 | 176.407 | 181.089 |
| pvdJ | Nutritional/Metabolic factor | 303.949 | 302.706 | 291.289 | 304.424 | 303.444 | 292.665 |
| KPR_RS09045 | Immune modulation | 235.522 | 225.365 | 227.209 | 236.142 | 232.419 | 241.312 |
| clpV | Effector delivery system | 167.972 | 165.611 | 157.637 | 175.887 | 164.039 | 167.384 |
| gacS | Regulation | 148.748 | 146.159 | 133.031 | 148.647 | 143.759 | 141.483 |
| hemC | Nutritional/Metabolic factor | 233.929 | 239.296 | 230.228 | 235.271 | 226.923 | 223.161 |
| ggt | Nutritional/Metabolic factor | 158.028 | 153.586 | 149.514 | 147.026 | 150.43 | 140.704 |
| hasA | Immune modulation | 162.322 | 154.654 | 165.035 | 167.848 | 153.169 | 157.773 |
| htrB | Immune modulation | 175.775 | 174.363 | 166.244 | 174.447 | 160.961 | 168.822 |
| PG_RS06845 | Nutritional/Metabolic factor | 448.854 | 438.53 | 433.285 | 447.508 | 441.295 | 443.089 |
| flrB | Motility | 174.819 | 181.01 | 163.856 | 174.607 | 169.599 | 171.51 |
| fecE | Nutritional/Metabolic factor | 236.244 | 237.684 | 233.661 | 248.072 | 231.712 | 235.534 |
| DDA3937_RS07715 | Nutritional/Metabolic factor | 183.232 | 187.784 | 182.223 | 196.921 | 187.723 | 193.537 |
| scpA/scpB | Adherence | 145.216 | 134.965 | 133.818 | 146.14 | 134.464 | 135.499 |
| fbpC | Nutritional/Metabolic factor | 341.013 | 333.536 | 326.299 | 341.894 | 334.699 | 336.725 |
| wbmC | Immune modulation | 149.651 | 143.573 | 149.806 | 145.277 | 159.696 | 149.319 |
| secA2 | Others | 183.885 | 176.698 | 178.348 | 189.469 | 180.595 | 189.849 |
| rpoN | Adherence | 162.797 | 149.349 | 154.083 | 158.088 | 155.779 | 147.816 |
| clbM | Exotoxin | 153.105 | 139.89 | 142.676 | 148.07 | 139.503 | 140.27 |
| LPG_RS06795 | Others | 126.481 | 130.358 | 132.206 | 128.555 | 123.609 | 117.248 |
| kpsF | Immune modulation | 152.963 | 158.678 | 161.264 | 150.953 | 163.882 | 152.017 |
| motD | Motility | 165.248 | 171.416 | 156.223 | 164.082 | 160.287 | 159.197 |
| ACICU_RS00395 | Immune modulation | 138.981 | 147.742 | 133.154 | 137.913 | 141.312 | 133.757 |
| YPO_RS16520 | Immune modulation | 134.239 | 134.039 | 133.055 | 140.355 | 124.087 | 136.222 |
| essC | Effector delivery system | 147.034 | 154.066 | 156.526 | 158.967 | 147.449 | 147.255 |
| rtxA | Exotoxin | 195.926 | 206.015 | 196.565 | 200.2 | 191.272 | 202.864 |
| htpB | Adherence | 191.903 | 198.543 | 196.582 | 201.06 | 197.736 | 186.503 |
| EAMY_RS32245 | Nutritional/Metabolic factor | 132.915 | 127.709 | 125.441 | 139.778 | 136.03 | 133.45 |
| hlyD | Exotoxin | 169.911 | 165.827 | 162.757 | 161.326 | 159.747 | 154.703 |
| PSYR_RS05455 | Exotoxin | 130.239 | 136.544 | 131.659 | 140.789 | 128.288 | 127.31 |
| cpsG | Immune modulation | 179.493 | 180.558 | 171.366 | 182.733 | 171.663 | 182.096 |
| glgX | Effector delivery system | 157.911 | 152.615 | 147.819 | 153.875 | 147.183 | 144.208 |
| mlsA2 | Exotoxin | 187.291 | 198.621 | 198.501 | 199.143 | 192.755 | 190.189 |
| lap | Adherence | 146.225 | 159.098 | 145.885 | 151.152 | 149.933 | 146.762 |
| vpsI | Biofilm | 188.75 | 188.572 | 179.031 | 183.863 | 179.255 | 177.596 |
| plcD | Exotoxin | 150.177 | 152.91 | 158.042 | 161.217 | 151.738 | 148.224 |
| YE105_RS07900 | Immune modulation | 218.515 | 215.3 | 205.092 | 211.385 | 216.524 | 210.391 |
| algB | Biofilm | 149.751 | 152.806 | 148.296 | 140.557 | 153.329 | 145.263 |
| KPK_RS08095 | Immune modulation | 162.167 | 161.713 | 155.598 | 156.214 | 149.051 | 155.334 |
| mmpL11 | Nutritional/Metabolic factor | 126.714 | 128.878 | 126.679 | 127.954 | 123.028 | 115.954 |
| QU43_RS45020 | Nutritional/Metabolic factor | 167.067 | 154.701 | 156.837 | 162.828 | 156.296 | 156.79 |
| bsh | Stress survival | 158.195 | 165.095 | 156.624 | 151.512 | 154.513 | 153.6 |
| pvdH | Nutritional/Metabolic factor | 142.598 | 137.556 | 141.968 | 141.22 | 134.878 | 130.709 |
| LPG_RS08320 | Effector delivery system | 162.475 | 159.885 | 155 | 160.606 | 161.821 | 150.466 |
| fliI | Motility | 177.766 | 182.755 | 179.11 | 185.303 | 190.763 | 182.205 |
| algI | Biofilm | 139.883 | 136.936 | 128.292 | 132.206 | 129.36 | 130.219 |
| wcbT | Immune modulation | 287.862 | 294.706 | 286.042 | 289.388 | 287.039 | 280.583 |
| PLU_RS18745 | Effector delivery system | 145.77 | 149.63 | 139.133 | 149.319 | 141.591 | 140.392 |
| recN | Stress survival | 195.991 | 196.952 | 189.725 | 201.027 | 190.202 | 198.189 |
| wbmI | Immune modulation | 162.566 | 159.683 | 165.48 | 157.047 | 152.717 | 161.533 |
| F7308_RS05575 | Immune modulation | 140.848 | 145.621 | 137.7 | 135.729 | 132.764 | 136.587 |
| mgtB | Nutritional/Metabolic factor | 145.012 | 143.405 | 137.347 | 137.562 | 140.508 | 132.869 |
| fauA | Nutritional/Metabolic factor | 153.624 | 143.626 | 149.499 | 154.846 | 149.91 | 145.221 |
| BCAH187_RS26550 | Immune modulation | 150.444 | 139.494 | 143.494 | 147.963 | 143.063 | 139.603 |
| hutA | Nutritional/Metabolic factor | 182.752 | 182.818 | 184.289 | 191.024 | 179.739 | 178.453 |
| KP1_RS17330 | Immune modulation | 139.802 | 144.172 | 138.356 | 149.506 | 139.027 | 145.01 |
| hgpB | Nutritional/Metabolic factor | 189.136 | 190.277 | 185.895 | 196.307 | 190.352 | 197.108 |
| lpxK | Immune modulation | 204.895 | 202.97 | 198.557 | 210.251 | 206.513 | 202.393 |
| piplc | Exotoxin | 167.124 | 167.936 | 165.725 | 172.913 | 174.329 | 174.631 |
| plc2 | Exotoxin | 126.755 | 134.741 | 124.736 | 134.012 | 131.042 | 130.955 |
| hutR | Nutritional/Metabolic factor | 142.719 | 144.929 | 140.162 | 143.7 | 135.059 | 136.981 |
| PMI_RS06920 | Nutritional/Metabolic factor | 117.949 | 120.962 | 116.288 | 123.086 | 118.009 | 111.88 |
| rhsP2 | Effector delivery system | 152.087 | 157.314 | 147.569 | 155.222 | 148.37 | 151.376 |
| qbsI | Nutritional/Metabolic factor | 132.229 | 128.802 | 129.957 | 126.163 | 122.105 | 124.913 |
| PSEEN_RS11610 | Nutritional/Metabolic factor | 163.019 | 160.303 | 165.732 | 169.735 | 162.89 | 167.691 |
| mbtI | Nutritional/Metabolic factor | 136.685 | 138.319 | 138.863 | 132.163 | 136.338 | 130.238 |
| ABB77403 | Effector delivery system | 178.511 | 177.872 | 180.181 | 179.168 | 177.583 | 171.976 |
| EAMY_RS19925 | Effector delivery system | 126.492 | 128.81 | 129.26 | 128.357 | 124.487 | 122.251 |
| fepC | Nutritional/Metabolic factor | 132.888 | 135.901 | 132.801 | 132.392 | 127.341 | 132.299 |
| proC | Nutritional/Metabolic factor | 176.025 | 175.993 | 182.877 | 178.297 | 179.864 | 180.818 |
| katG | Stress survival | 136.689 | 137.404 | 134.752 | 131.819 | 134.131 | 130.854 |
| BJAB07104_RS00505 | Immune modulation | 198.646 | 203.954 | 199.198 | 201.967 | 200.971 | 203.564 |
| pgaC | Biofilm | 120.118 | 118.379 | 117.158 | 118.545 | 117.21 | 116.635 |
